# Shade compromises the photosynthetic efficiency of NADP-ME less than that of PEP-CK and NAD-ME C_4_ grasses

**DOI:** 10.1093/jxb/ery129

**Published:** 2018-04-05

**Authors:** Balasaheb V Sonawane, Robert E Sharwood, Spencer Whitney, Oula Ghannoum

**Affiliations:** 1 ARC Centre of Excellence for Translational Photosynthesis and Hawkesbury Institute for the Environment, Western Sydney University, NSW, Australia; 2 School of Biological Sciences, Washington State University, Pullman, WA, USA; 3 ARC Centre of Excellence for Translational Photosynthesis and Research School of Biology, Australian National University, Canberra, ACT, Australia

**Keywords:** Biochemical subtypes, C_4_ photosynthesis, CO_2_-concentrating mechanism, low light, shade

## Abstract

The high energy cost and apparently low plasticity of C_4_ photosynthesis compared with C_3_ photosynthesis may limit the productivity and distribution of C_4_ plants in low light (LL) environments. C_4_ photosynthesis evolved numerous times, but it remains unclear how different biochemical subtypes perform under LL. We grew eight C_4_ grasses belonging to three biochemical subtypes [NADP-malic enzyme (NADP-ME), NAD-malic enzyme (NAD-ME), and phosphoenolpyruvate carboxykinase (PEP-CK)] under shade (16% sunlight) or control (full sunlight) conditions and measured their photosynthetic characteristics at both low and high light. We show for the first time that LL (during measurement or growth) compromised the CO_2_-concentrating mechanism (CCM) to a greater extent in NAD-ME than in PEP-CK or NADP-ME C_4_ grasses by virtue of a greater increase in carbon isotope discrimination (∆_P_) and bundle sheath CO_2_ leakiness (ϕ), and a greater reduction in photosynthetic quantum yield (Φ_max_). These responses were partly explained by changes in the ratios of phosphoenolpyruvate carboxylase (PEPC)/initial Rubisco activity and dark respiration/photosynthesis (*R*_d_/*A*). Shade induced a greater photosynthetic acclimation in NAD-ME than in NADP-ME and PEP-CK species due to a greater Rubisco deactivation. Shade also reduced plant dry mass to a greater extent in NAD-ME and PEP-CK relative to NADP-ME grasses. In conclusion, LL compromised the co-ordination of the C_4_ and C_3_ cycles and, hence, the efficiency of the CCM to a greater extent in NAD-ME than in PEP-CK species, while CCM efficiency was less impacted by LL in NADP-ME species. Consequently, NADP-ME species are more efficient at LL, which could explain their agronomic and ecological dominance relative to other C_4_ grasses.

## Teaser

Photosynthesis and productivity are less compromised by low light in NADP-ME C_4_ grasses relative to PEP-CK and NAD-ME counterparts.

## Introduction

C_4_ photosynthesis is characterized by the operation of a CO_2_-concentrating mechanism (CCM) whereby atmospheric CO_2_ is initially fixed in the mesophyll cells (MCs) into C_4_ acids. These acids are subsequently decarboxylated in the bundle sheath cells (BSCs) releasing CO_2_ where Rubisco, the ultimate CO_2_-fixing enzyme, is located (Hatch, 1987). The CCM serves to raise the CO_2_ concentration in the BSCs, thus curbing photorespiration and CO_2_-saturating photosynthesis at current ambient CO_2_ concentrations ([CO_2_]) under high light (Hatch, 1987; Kanai and Edwards, 1999). The CCM requires additional energy costs compared with the C_3_ cycle associated with the regeneration of the C_3_ precursor phosphoenolpyruvate (PEP) and the overcycling of CO_2_. Nevertheless, under warm temperatures, C_4_ plants have a superior photosynthetic quantum yield (Ф_max_) relative to C_3_ plants (Ehleringer and Björkman, 1977; Pearcy *et al.*, 1981; Ehleringer and Pearcy, 1983; Zhu *et al.*, 2008). This explains the ecological dominance of C_4_ plants in open, high light (HL) environments and their disproportionately high global productivity relative to their small taxonomic representation (Ehleringer *et al.*, 1997; Brown, 1999; Edwards *et al.*, 2010).

Despite their success under HL, C_4_ plants experience low light (LL) under natural conditions. C_4_ crops and grasses can form dense canopies where a significant proportion of the leaf area is shaded, in addition to short-term LL exposures during the course of the day (Sage, 2014; Ort *et al.*, 2015). Numerous C_4_ grasses are adapted to the shade of the forest interior (Sage and Pearcy, 2000). Shading is expected to increase in the understorey of C_4_ grass-dominated ecosystems with predicted woody thickening under increasing atmospheric [CO_2_] (Bond and Midgley, 2012; Saintilan and Rogers, 2015). Consequently, it is important to investigate the efficiency of C_4_ photosynthesis under LL across diverse C_4_ plants.

C_4_ photosynthesis has evolved independently many times, resulting in three biochemical subtypes named after the primary C_4_ acid decarboxylase enzyme found in the BSCs, and they are NADP-malic enzyme (ME), NAD-ME, and phosphoenolpyruvate carboxykinase (PEP-CK) (Hatch 1987). PEP-CK operates as a secondary decarboxylase in many C_4_ species (Leegood and Walker, 2003; Furbank, 2011; Sharwood *et al.*, 2014; Wang *et al.*, 2014). However, the primary decarboxylase is generally associated with a suite of anatomical, biochemical, and physiological features (Gutierrez *et al.*, 1974; Hattersley, 1992; Kanai and Edwards, 1999; Ghannoum *et al.*, 2011), making it a suitable classification basis for the purpose of the current study investigating the efficiency of the C_4_-CCM. The grass family includes species from all biochemical subtypes (Sage and Pearcy, 2000; Edwards *et al.*, 2010), but our understanding of how different C_4_ subtypes respond and acclimate to LL environments remains limited.

It is well demonstrated that the C_4_ subtypes have different leaf dry matter carbon isotope composition (δ^13^C) and Ф_max_. In particular, C_4_ grasses with the NAD-ME subtype (i.e. NAD-ME as the primary decarboxylase) show lower leaf δ^13^C (i.e. more negative values which are closer to the C_3_ range) and Ф_max_, while NADP-ME and PEP-CK species (i.e. those with NADP-ME and PEP-CK as the primary decarboxylases, respectively) show the highest and intermediate values, respectively (Hattersley, 1982; Ehleringer and Pearcy, 1983). There is a paucity of data comparing the response to shade of leaf δ^13^C and Ф_max_ of the various C_4_ subtypes. Using 14 C_4_ grasses, Buchmann *et al.* (1996) found that leaf δ^13^C values of NAD-ME species were most impacted by shade, followed by PEP-CK and NADP-ME species. In a large survey of C_4_ grasses, von Caemmerer *et al.* (2014) reported that leaf δ^13^C was equally affected by growing season irradiance (winter versus summer) in NAD-ME and NADP-ME grasses. This discrepancy may be due to the fact that carbon isotope composition and discrimination are not significantly affected until photosynthetic photon flux density (PPFD) decreases below 700 μmol m^–2^ s^–1^ (Buchmann *et al.*, 1996).

In C_4_ plants, both photosynthetic and post-photosynthetic discrimination factors determine leaf δ^13^C (Farquhar, 1983; von Caemmerer *et al.*, 2014). Reduced leaf δ^13^C under LL may be attributed to increased photosynthetic carbon isotope discrimination (Δ_P_) and BSC leakiness (ϕ) as a result of reduced CCM efficiency (Henderson *et al.*, 1992). For example, LL may reduce the activity of the C_3_ cycle to a greater extent than that of the C_4_ cycle, leading to greater overcycling. In turn, this will lead to higher ϕ and energetic cost of operating the C_4_-CCM. Consequently, we hypothesized that LL will differentially compromise the C_4_-CCM efficiency depending on the biochemical subtype. In particular, we predicted that LL will increase leaf δ^13^C and Δ_P_, and reduce Ф_max_ to a greater extent in NAD-ME grasses, followed by PEP-CK and NADP-ME species (Hypothesis 1).

Short- and long-term photosynthetic responses to LL are expected to differ. Following long-term exposure to LL (shade), the photosynthetic apparatus commonly acclimates to maximize light use efficiency (Björkman, 1981; Boardman, 1977; Sage and McKown, 2006). Depending on the plant species and ecotype, acclimation may be minimal or profound (Ward and Woolhouse, 1986*a*, *b*; Björkman and Holmgren, 1966). Overall, acclimation of C_3_ and C_4_ plants to shade involves partitioning of photosynthetic nitrogen (N) away from Rubisco towards the light-harvesting processes (Boardman, 1977; Björkman, 1981; Hikosaka and Terashima, 1995; Evans and Poorter, 2001; Walters, 2005; Tazoe *et al.*, 2006; Pengelly *et al.*, 2010). NAD-ME species are known to have higher leaf N content and a greater N fraction invested in Rubisco relative to NADP-ME species (Ghannoum *et al.*, 2005). Hence, the former subtype may have a greater flexibility to reallocate N under shade especially since, as mentioned above, LL is expected to reduce the activity of the C_3_ cycle (e.g. Rubisco) more than the C_4_ cycle [e.g. phosphoenolpyruvate carboxylase (PEPC)]. However, optimal photosynthetic acclimation of C_4_ photosynthesis to shade is expected to involve parallel reductions in the activities of the C_3_ and C_4_ cycles in order to curb leakiness and maintain an efficient CCM and quantum yield (Bellasio and Griffiths, 2014*a*, *b*), which does not seem to be the case for NAD-ME species in Buchmann *et al.* (1996). Consequently, we hypothesized that NAD-ME species will exhibit a greater photosynthetic acclimation in response to shade relative to the other two subtypes. This will manifest as a greater photosynthetic down-regulation and higher leakiness in the LL-acclimated leaves of NAD-ME species relative to the other two subtypes (Hypothesis 2).

To address these two hypotheses, we investigated the photosynthetic responses of eight C_4_ grasses belonging to three biochemical subtypes (Table 1) to short-term (200 µmol quanta m^–2^ s^–1^ versus 2000 µmol quanta m^–2^ s^–1^) and long-term (16% versus 100% sunlight; Supplementray Fig. S1A at *JXB* online) light treatments. We sought to elucidate the underlying mechanisms by describing changes in photosynthetic rates and enzyme activities, and in the CCM efficiency as described by leakiness and quantum yield. Our results indicated that NADP-ME species are generally more efficient at LL due to effective co-ordination of the C_4_ and C_3_ cycles.

**Table 1.** List of C_4_ grasses used in the current study

C_4_ subtype	C_4_ tribe	Species
NADP-ME	Paniceae	*Cenchrus ciliaris* *Panicum antidotale*
	Andropogoneae	*Sorghum bicolor* *Zea mays*
PEP-CK	Paniceae Chloridoideae	*Megathyrsus maximus* *Chloris gayana*
NAD-ME	Paniceae Chloridoideae	*Panicum coloratum* *Leptochloa fusca*

## Materials and methods

### Plant culture

The experiment was conducted in a naturally lit glasshouse chamber (5 m^3^) during the Australian summer. Within the chamber, an aluminium structure (1.5 × 5 m^3^) was covered with white shade cloth (Premium Hortshade Light, Model No. 428976, Coolaroo, VIC, Australia). A PPFD of ~100 μmol quanta m^–2^ s^–1^ was achieved inside the shade structure by adjusting the number of cloth layers. The impact of heavy shade during cloudy days was minimized by supplementing external growth light (LimiGrow Pro S325, Emeryville, CA, USA) to achieve a leaf-level PPFD of 100 μmol m^–2^ s^–1^. The chamber temperature was maintained at 28/22 °C for day/night with an in-built glasshouse temperature-controlled system. The air temperature and relative humidity (RH) at leaf level were monitored using Rotronic HC2-S3 (Bassersdorf, Switzerland) sensors placed in a shield vented with a 12 V fan. A Licor quantum sensor (LI-190, Lincoln, NE, USA) was mounted at the leaf level to monitor incident PPFD in the unshaded and shaded glasshouse structure. Data from these sensors were stored using a Licor data logger (LI-1400). Through the experiment, average midday ambient PPFD, *T*, and RH at leaf level were 741 μmol m^–2^ s^–1^, 25 ºC, and 69% in the sun treatment. These figures in the shade treatment were 119 μmol m^–2^ s^–1^, 26 °C, and 65% (see Supplementary Fig. S1C). Hence, the shade treatment was equivalent to 16% of sunlight measured in the sun treatment, averaged over the experimental period (Supplementary Fig. S1). Instantaneous leaf temperature was measured using a hand-held, non-contact infrared thermometer (AGRI-THERM II™, Chino Hills, CA USA). On average, shaded leaves were 1–2 °C cooler than sun leaves (Supplementary Fig. S1D).

Locally collected soil was sun dried (Pinto *et al.*, 2014), coarsely sieved, and added to 3.5 litre cylindrical pots. Pots were watered to 100% capacity and transferred to the glasshouse chamber. Seeds for grasses used in this study (Table 1) were obtained from the Australian Plant Genetic Resources Information System (QLD, Australia) and Queensland Agricultural Seeds Pty. Ltd. (Toowoomba, Australia). In the current study, we used 2–3 representative species belonging to each of the C_4_ subtypes. Within each subtype, species were selected from different C_4_ origins (tribes in Table 1) to randomize the C_4_ origin effect, and hence focus on the subtype effect.

Seeds were germinated in a commercial Osmocote^®^ professional, seed raising and cutting mix (Scotts, Bella Vista, NSW, Australia). Three to four weeks after germination, two healthy seedlings were transplanted into each of the soil-filled and pre-irrigated pots. A week later, one healthy seedling was left in each pot while the other was removed. Plants were allowed to grow until the 5–6 leaf stage in full sunlight before they were transferred to the shade treatment. There were eight pots per species and light treatment. Pots were randomly positioned and regularly rotated within each treatment throughout the experiment. Plants were well watered daily with added commercial soluble fertilizer (Aquasol, N:P:K=23.3:3.95:14; Yates, Wetherill Park, NSW, Australia).

### Leaf gas exchange measurements

Leaf gas exchange was measured with a portable open gas exchange system (LI-6400XT, LI-COR). The youngest last fully expanded leaf (LFEL) on the main stem of a 6- to 9-week-old plant was measured at a leaf temperature of 28 °C between 10.00 h and 14.00 h. Shaded plants developed a minimum of three new leaves under shade before gas exchange measurements were made.

Each leaf was allowed to reach a steady state, at least 20 min, CO_2_ assimilation rate (*A*) at ambient [CO_2_] of 407 µbar, PPFD of 2000 μmol m^–2^ s^–1^, and RH of 50–70%. After this, a steady-state measurement [performed concurrently with tunable diode-laser (TDL) analysis; see below for details] was taken. Subsequently, the response of *A* to step increases of intercellular CO_2_ (*C*_i_), the *A*–*C*_i_ curve, was measured by raising the LI-6400XT leaf chamber [CO_2_] in 10 steps between 50 µbar and 1500 µbar. After completing the *A*–*C*_i_ curve at PPFD 2000 μmol m^–2^ s^–1^, the leaf was allowed to reach a steady state of gas exchange at saturating CO_2_ (660 µbar) before measuring the responses to PPFD. The light response curve was measured from HL to LL (11 steps) followed by measurements of dark respiration (*R*_d_) at ambient [CO_2_] of 407 µbar after 20 min in a dark leaf chamber. Prior to LL steady-state measurement (performed concurrently with TDL analysis; see below for details), the same leaf was allowed to reach a steady-state *A* similarly to HL measurements, except PPFD was controlled at 250 μmol m^–2^ s^–1^. This was followed by measuring the *A*–*C*_i_ curve at the same light (LL) as described above. There were 3–4 replicates per treatment. The initial slope (IS) of the *A*–*C*_i_ curve was estimated for the linear part of the *A*–*C*_i_ curve measured at HL where *C*_i_ is <55 µbar. The aximum CO_2_ assimilation rate (*A*) on the *A*–*C*_i_ curve was considered as the CO_2_-saturated rate (CSR). The *A*–*C*_i_ curves measured at LL could not be used for accurate IS determination due to low overall rates. The light response curves were fitted using the following equation (Ögren and Evans, 1993):

$$A=\frac{(\Phi_{\text{nls}}\cdot I+A_{\text{max}})-\sqrt{{\left(\Phi_{\text{nls}}\cdot I+A_{\text{max}}\right)}^{2}-4\cdot \theta \cdot I\cdot A_{\text{max}}}}{2\cdot \theta }$$ (1)

where, *I*=absorbed irradiance, we assumed absorptance=0.85; *A*=CO_2_ assimilation rate at given light; Φ_nls_=maximum quantum yield of PSII; *A*_max_=light-saturated CO_2_ assimilation rate; and θ=curvature factor of the light response curve. In addition, the slope of a linear part of the light response curve (PPFD <120 μmol m^–2^ s^–1^) was estimated as the ‘apparent’ maximum quantum yield of PSII (Ф_max_). We consider this estimate in our further analysis.

### Photosynthetic carbon isotope discrimination

Bundle sheath leakiness (ϕ) was determined by measuring real-time ^13^CO_2_/^12^CO_2_ isotope discrimination using a LI-6400XT interfaced with a tunable diode laser, TDL (TGA100, Campbell Scientific, Inc., Logan, UT, USA). The mean SD for repeated TDL measurements of δ^13^C values for a reference gas was 0.09‰. Observed photosynthetic carbon isotope discrimination against (Δ_P_) was calculated using (Evans *et al.*, 1986):

$$\Delta_{P}=\frac{\xi \cdot (\delta_{\text{o}}-\delta_{\text{e}})}{1+\delta_{\text{o}}-\xi \cdot (\delta_{\text{o}}-\delta_{\text{e}})}$$ (2)

$$\xi =\;\frac{C_{\text{e}}}{C_{\text{e}}-C_{\text{o}}}$$ (3)

where δ_e_, δ_o_, *C*_e_, and *C*_o_ are the δ^13^C (δ) and CO_2_ mole fraction (*C*) of the air entering (e) and leaving (o) the leaf chamber measured with the TDL-LI-6400 set up. Leakiness at high light (ϕ_h_) was calculated using the model of Farquhar (1983) as modified by Pengelly *et al.* (2010, 2012) and von Caemmerer *et al.* (2014):

$$\phi_{h}=\frac{\left(\frac{1-t}{1+t}\right)\cdot \Delta_{\text{p}}-\frac{a^{\text{'}}}{1+t\;}-\left(a_{i}-b_{4}^{\text{'}}\right)\cdot \frac{A}{g_{\text{m}}\cdot C_{\text{a}}}-\left(b_{4}^{\text{'}}-\frac{a^{\text{'}}}{1+t}\;\right)\cdot \frac{C_{\text{i}}}{C_{\text{a}}}}{(b_{3}^{\text{'}}-s)\cdot \left(\frac{C_{i}}{C_{a}}-\frac{A}{C_{a}\cdot g_{m}}\right)}$$ (4)

where *t*, the ternary correction factor, is calculated as per Farquhar and Cernusak (2012):

$$t=\;\frac{(1+a^{'})\cdot E}{2\cdot g_{\text{ac}}^{t}},$$ (5)

where *E* is the transpiration rate, *g*^t^_ac_ the total conductance to CO_2_ diffusion including boundary layer and stomatal conductance (von Caemmerer and Farquhar, 1981). The combined fractionation factor through the leaf boundary layer and stomata is denoted by *a*′,

$$a'=\frac{a_{\text{b}\cdot }(C_{\text{a}}-\;C_{\text{ls}})+\;a\cdot (C_{\text{ls}}-C_{\text{i}})}{C_{\text{a}}-C_{\text{i}}}$$ (6)

Definition and units for the variables included in the above equation are described in Table 2, but briefly, *C*_a_, *C*_i_, and *C*_ls_ are the ambient, intercellular and leaf surface CO_2_ mole fractions respectively; *a*_b_ (2.9‰) is the fractionation occurring through diffusion in the boundary layer; *s* (1.8‰) is the fractionation during leakage of CO_2_ out of the bundle sheath assuming there is no HCO_3_^–^ leakage out of BSCs (Henderson *et al.*, 1992); *a* (4.4‰) is the fractionation due to diffusion in air (Evans *et al.*, 1986); and *a*_i_ is the fractionation factor associated with the dissolution of CO_2_ and diffusion through water (1.8‰).

**Table 2.** Statistical summary

Parameter	Species	Treat	Species×treatment	Species (rand)	Subtype	Treatment	Subtype×treatment
Total DM (g per plant)	***	***	***	***	*	**	**
Total leaf area (m^2^ per plant)	***	***	***	***	ns	*	*
Root/shoot DM	***	***	***	***	ns	**	ns
LMA (g m^–2^)	***	***	***	***	ns	*	ns
Leaf N_mass_ (mg g^–1^)	***	*	***	***	ns	ns	ns
Leaf N_area_ (gm^–2^)	***	***	***	**	ns	***	ns
Leaf NUE	***	***	**	***	0.10	**	*
∆_DM_ (‰)	***	***	***	***	*	*	*
PNUE (µmol CO_2_ s^–1^ g^–1^ N)	***	***	***	NA	0.10	0.07	0.06
PWUE (µmol CO_2_ mol^–1^ H_2_O)	***	*	*	***	ns	ns	ns
*A*_h_ at HL (µmol m^–2^ s^–1^)	***	***	***	***	0.09	**	*
*A*_l_ at LL (µmol m^–2^ s^–1^)	***	**	***	**	ns	*	*
*g*_sh_ at HL (µmol m^–2^ s^–1^)	***	***	**	***	ns	**	0.07
*g*_sl_ at LL (µmol m^–2^ s^–1^)	*	ns	ns	*	ns	ns	ns
∆_Ph_ at HL (‰)	***	***	*	***	ns	0.10	ns
∆_Pl_ at LL (‰)	***	ns	ns	***	ns	ns	ns
*C*_i_/*C*_ah_ at HL	***	***	0.06	***	ns	ns	ns
*C*_i_/*C*_al_ at LL	***	ns	*	0.07	ns	ns	ns
Leakiness (Ф_h_) at HL	***	**	*	***	ns	**	ns
Leakiness (Ф_l_) at LL	***	ns	ns	***	ns	ns	ns
*R*_d_ (µmol m^–2^ s^–1^)	***	***	*	**	ns	0.06	ns
IS at HL (µmol m^–2^ s^–1^ bar^–1^)	***	***	***	***	ns	*	0.10
CSR at HL (µmol m^–2^ s^–1^)	***	***	***	***	0.07	**	*
IS/CSR at HL	***	ns	*	*	ns	ns	ns
*A*_max_ (µmol m^–2^ s^–1^)	***	***	***	NA	0.10	**	*
Фmax (mol CO2 mol-1quanta)	***	***	***	***	0.08	*	*
Curvature factor (θ)	***	0.08	***	*	0.10	0.08	*
Initial Rubisco activity	*	***	*	*	ns	*	*
Rubisco activity (µmol m^–2^ s^–1^)	ns	***	ns	*	ns	*	ns
Rubisco sites (µmol m^–2^)	***	***	***	***	ns	*	ns
Rubisco activation (%)	***	***	***	*	ns	0.10	0.07
PEPC activity (µmol m^–2^ s^–1^)	***	***	***	***	0.10	**	*
PEPC/Rubisco activity	***	***	***	***	0.08	*	*
NADP-ME activity (µmol m^–2^ s^–1^)	***	***	***	NA	*	*	*
NAD-ME activity (µmol m^–2^ s^–1^)	***	***	***	***	0.10	*	ns
PEP-CK activity (µmol m^–2^ s^–1^)	***	***	***	***	ns	*	ns
DCs (µmol m^–2^ s^–1^)	***	***	***	***	ns	*	0.10
Protein (g m^–2^)	*	***	***	NA	ns	*	ns

Summary of statistical analysis using two-way ANOVA to test for the effects of species and light treatment, and a linear-mixed effect model to test subtype and light treatment effects where species were considered as a random variable.

ns, not significant (*P*>0.05); *P<0.05; ** *P*<0.01; ***P<0.001

$b_{3}^{'}$ and $b_{4}^{'}$ are defined as in von Caemmerer *et al.* (2014):

$$b_{3}^{'}=\;b_{3}-e\cdot \left(\frac{R_{d}}{A+R_{d}}-\frac{0.5\cdot R_{d}}{A+0.5\cdot R_{d}}\right)-f\cdot \frac{0.5\cdot V_{0}}{V_{\text{c}}},$$ (7)

and

$$b_{4}^{'}=b_{4}-e\cdot \frac{\;0.5\;\cdot R_{\text{d}}}{\left(A+0.5\cdot R_{\text{d}}\right)}$$ (8)

where *b*_3_ is the fractionation by Rubisco (30‰); *b*_4_ is the combined fractionation of the conversion of CO_2_ to HCO_3_^–^ and PEP carboxylation (–5.41‰ at 28 °C)(Henderson *et al.*, 1992; Mook *et al.*, 1974); *f* is the fraction associated with photorespiration; and *V*_o_ and *V*_c_ are the rates of oxygenation and carboxylation, respectively. Under HL we assumed no photorespiration, hence the term $f\cdot \frac{0.5\cdot V_{0}}{V_{\text{c}}}=0$ (Pengelly *et al.*, 2010, 2012; Ubierna *et al.*, 2013; von Caemmerer *et al.*, 2014). The reference gas supplied to the LI-6400XT during gas exchange measurements had δ^13^C= –5.5‰. Therefore, the value for the fractionation factor *e* associated with respiration was calculated assuming recent photoassimilates as the respiratory substrate (Stutz *et al.*, 2014). Thus, *e* equalled the difference between δ^13^C in the CO_2_ sample line in LI-6400XT and that in the glasshouse chamber (–8‰; Tazoe *et al.* (2008)). *A* and *R*_d_ (von Caemmerer *et al.*, 2014) denote the CO_2_ assimilation rate and day respiration, respectively; *R*_d_ was assumed to equal measured dark respiration. We assumed mesophyll conductance, *g*_m_=1.4 mol m^–2^ s^–1^ at 28 °C (for C_4_ the model plant *Setaria viridis*) (Ubierna *et al.*, 2017).

Leakiness at low light (ϕ_l_) was calculated as described by Bellasio and Griffiths (2014*b*) and Ubierna *et al.* (2013). Briefly, electron transport flux (*J*_t_) at low light was derived by deploying the light-limited C_4_ photosynthesis model to calculate *C*_s_ (CO_2_ mole fraction in the bundle sheath), *V*_p_ (PEP carboxylation rate), *V*_c_ (Rubisco carboxylation rate), and *V*_o_ (Rubisco oxygenation rate) at LL using the C_4_ model (von Caemmerer, 2000) (see Supplementary Appendix S1). It should be noted that we used measured values for the fraction of PSII in BSCs, α (0 for NADP-ME and 0.2 for PEP-CK and NAD-ME) and half of the reciprocal of Rubisco specificity, γ* (0.000255, 0.00023, and 0.000233 for NADP-ME, NAD-ME, and PEP-CK, respectively) (Table 2; Sharwood *et al.*, 2016*a*) for biochemical subtypes of C_4_ photosynthesis during the calculation of *J*_t_. These parameters were then used to calculate $\bar{b_{4}}$ (the combined effects of fractionations by the CO_2_ dissolution, hydration, and PEPC activity at LL) and $\bar{b_{3}}$ (Rubisco fractionation at LL by accounting for the fraction during respiration and photorespiration) (Farquhar, 1983; Ubierna *et al.*, 2013) to calculate ϕ_l_ in the following equation,

$$\phi_{\text{l}}=\left(\frac{C_{bs}-C_{m}}{C_{m}}\right)\cdot \frac{\Delta_{\text{p}}\cdot \left(1-t\right)\cdot C_{a}-a^{'}\cdot \left(C_{a}-C_{i}\right)-a_{i}\cdot \left(C_{i}-C_{m}\right)\cdot \left(1+t\right)-\left(1+t\right)\cdot C_{m}\cdot \bar{b_{4}}}{\left(1+t\right)\cdot \left[\bar{b_{3}}\cdot C_{bs}-s\cdot \left(C_{bs}-C_{m}\right)+\;a_{i}\cdot (C_{i}-C_{m})\right]+a^{'}\cdot \left(C_{a}-C_{i}\right)-C_{a}\cdot \Delta_{\text{p}}\cdot \left(1-t\right)}$$ (9)

other variables and unit are as defined in Table 2, but briefly *C*_m_ is the mesophyll CO_2_ mole fraction given by

$$C_{m}=\;C_{i}-\frac{A}{g_{m}}$$ (10)

Subtype-specific values for α and γ* improved the estimations of ϕ_l_ as demonstrated in Supplementary Fig. S7.

### Activity of Rubisco, PEPC, NADP-ME, NAD-ME, and PCK

Following gas exchange measurements, replicate discs (0.4–1 cm^2^) were rapidly frozen in liquid N then stored at –80 °C until analysed. Two sets of extractions were performed to complete the biochemical analysis. For Rubisco activity, activation, and content, PEPC and NADP-ME activity, and soluble protein assays, the extraction buffer was purged of CO_2_ overnight by bubbling a weak jet of nitrogen gas through the basic buffer [50 mM EPPS-NaOH (pH 7.8), 5 mM MgCl_2_, 1 mM EDTA]. Each leaf disc was extracted in 0.8 ml of ice-cold extraction buffer [50 mM EPPS-NaOH (pH 7.8), 5 mM DTT, 5 mM MgCl_2_, 1 mM EDTA, 10 μl of protease inhibitor cocktail (Sigma), 1% (w/v) polyvinyl polypyrrolidone (PVPP)] using a 2 ml Tenbroeck glass homogenizer kept on ice. Chlorophyll content was estimated according to Porra *et al.* (1989) by mixing 100 µl of total extract with 900 µl of acetone. The extract was then centrifuged at 15 000 *g* for 1 min and the supernatant was used for the subsequent assays. For Rubisco content, subsamples of the supernatant were incubated for 10 min in activation buffer [50 mM EPPS (pH 8.0), 10 mM MgCl_2_, 2 mM EDTA, 20 mM NaHCO_3_]. Rubisco content was estimated by the irreversible binding of [^14^C]CABP (2-C-carboxyarabinitol 1,5-bisphosphate) to the fully carbamylated enzyme (Sharwood *et al.*, 2008). Extractable soluble proteins were measured using the Pierce Coomassie Plus (Bradford) protein assay kit (Thermo Scientific, Rockford, IL, USA).

The activities of the photosynthetic enzymes Rubisco, PEPC, and NADP-ME were measured using spectrophotometric assays as described previously (Jenkins *et al.*, 1987; Ashton *et al.*, 1990; Sharwood *et al.*, 2008, 2014, 2016*b*; Pengelly *et al.*, 2010). Briefly, initial Rubisco activity was measured in assay buffer containing 50 mM EPPS-NaOH (pH 8), 10 mM MgCl_2_, 0.5 mM EDTA, 1 mM ATP, 5 mM phosphocreatine, 20 mM NaHCO_3_, 0.2 mM NADH, 50 U of creatine phosphokinase, 0.2 mg of carbonic anhydrase, 50 U of 3-phosphoglycerate kinase, 40 U of glyceraldehyde-3-phosphate dehydrogenase, 113 U of triose-phosphate isomerase, and 39 U of glycerol-3-phosphate dehydrogenase, and the reaction initiated by the addition of 0.22 mM ribulose-1,5-bisphosphate (RuBP). For maximal Rubisco activity, the supernatant was activated in assay buffer for 10 min at 25 °C before initiation of the reaction. Rubisco activation was calculated as the initial/maximal Rubisco activity ratio. Our Rubisco activity from *in vitro* assays was slightly lower than CO_2_ assimilation rates. Hence, in the current study, we presented Rubisco activity estimated from Rubisco sites measured with CABP assay and published Rubisco *K*_cat_ for individual species (Sharwood *et al.*, 2016*a*), and Rubisco activation values are from *in vitro* initial and activated Rubisco assays.

PEPC activity was measured in assay buffer [50 mM EPPS-NaOH (pH 8.0), 0.5 mM EDTA, 10 mM MgCl_2_, 0.2 mM NADH, 5 mM glucose-6-phosphate, 0.2 mM NADH, 1 mM NaHCO_3_, 1 U of malate dehydrogenase (MDH)] after the addition of 4 mM PEP. NADP-ME activity was measured in assay buffer [50 mM NADP-ME buffer (pH 8.3), 4 mM MgCl_2_, 0.5 mM NADP, 0.1 mM EDTA] after the addition of 5 mM malic acid.

The activity of PEP-CK was measured in the carboxylation direction using the method outlined previously (Koteyeva *et al.*, 2015; Sharwood *et al.*, 2016*b*). For PEP-CK and NAD-ME activity, a separate leaf disc was homogenized in extraction buffer containing 50 mM HEPES (pH 7.5), 2 mM EDTA, 0.05% Triton, 5 mM DTT, 1% PVPP, and 2 mM MnCl_2_ using a 2 ml Tenbroeck glass homogenizer kept on ice. The extract was centrifuged at 21 130 *g* for 1 min and the supernatant used for PEP-CK and NAD-ME activity assays. PEP-CK activity was measured in assay buffer containing 50 mM HEPS (pH 6.3), 4% β-mercaptoethanol, 100 mM KCl, 90 mM KHCO_3_, 0.5 mM ADP, 2 mM MnCl_2_, 0.2 mM NADH, 6 U of MDH, and 5 mM aspartic acid after the addition of 10 mM PEP. NAD-ME activity was measured in 25 mM Tricine (pH 8.3), 5 mM DTT, 2 mM NAD, 0.1 mM acetyl-CoA, 4 mM MnCl_2_, and 2 mM EDTA after the addition of 5 mM malic acid. Enzyme activity was calculated by monitoring the decrease/increase of NADH^+^ absorbance at 340 nm with a UV-VIS spectrophotometer (model 8453, Agilent Technologies Australia, Mulgrave, Victoria).

### SDS–PAGE and immunoblot analysis of photosynthetic proteins

SDS–PAGE and immunoblot analysis of photosynthetic proteins were performed as described in Sharwood *et al.* (2014). The procedures are described below.

Subsamples of total leaf extracts used for enzyme assays were mixed with 0.25 vols of 4× LDS buffer (Invitrogen) containing 100 mM DTT and placed in liquid nitrogen, then stored at –20 °C until they were analysed. For confirmatory visualization, protein samples were separated by SDS–PAGE in TGX Any kD (BioRad) pre-cast polyacrylamide gels buffered with 1× Tris-glycine SDS buffer (BioRad) at 200 V using the Mini-Protean apparatus at 4 °C. Proteins were visualized by staining with Bio-Safe Coomassie G-250 (BioRad) and imaged using the VersaDoc imaging system (BioRad).

For immunoblot analyses, samples of total leaf proteins were separated by SDS–PAGE as outlined above, then transferred at 4 °C to nitrocellulose membranes (0.45 µm; BioRad) using the Xcell Surelock western transfer module (Invitrogen) buffered with 1× Transfer buffer [20 × 25 mM Bicine, 25 mM Bis-Tris, 1 mM EDTA, 20% (v/v) methanol]. After 1 h transfer at 30 V, the membrane was placed in blocking solution [3% (w/v) skim milk powder in Tris-buffered saline (TBS); 50 mM Tris–HCl pH 8, 150 mM NaCl] for 1 h at room temperature with gentle agitation.

Primary antisera raised in rabbit against tobacco Rubisco (prepared by S.M. Whitney) was diluted 1:4000 in TBS before incubation for 1 h with membranes at room temperature with gentle agitation. Antiserum raised against PEPC (Cat. AS09 458) was obtained from AgriSera and diluted 1:2000 with TBS. For NADP-ME and PEP-CK, synthetic peptides based on monocot amino acid sequences for each protein were synthesized by GL Biochem and antisera were raised against each peptide in rabbits. The reactive antiserum was the antigen purified for use in immunoblot analysis (GL Biochem). The NADP-ME and PEP-CK antisera were diluted in TBS 1:1000 and 1:500, respectively. All primary antisera were incubated with membranes at room temperature for 1 h with gentle agitation before washing three times with TBS. Secondary goat anti-rabbit antiserum conjugated to horseradish peroxidase (HRP; Cat. NEF 812001EA, Perkin Elmer) was diluted 1:3000 in TBS and incubated with the membranes for 1 h at room temperature followed by three washes with TBS. Immunoreactive peptides were detected using the Immun-Star Western C kit (Cat. 170-5070, BioRad) and imaged using the VersaDoc.

### Leaf nitrogen and carbon isotope analyses

Following gas exchange and leaf disc sampling, the remainder of the LFEL was cut and its area was measured using a leaf area meter (LI-3100A, LI-COR). The LFEL was oven-dried, weighed, then milled to a fine powder. Leaf N content was determined on the ground leaf tissue samples using a CHN analyser (LECO TruSpec, LECO Corp., MI, USA). Leaf mass per area (LMA, g m^–2^) was calculated as total leaf dry mass/total leaf area. Leaf N per unit area (N_area_) was calculated as (mmol N g^–1^)×LMA (g m^–2^). For leaf δ^13^C, ground leaf samples were combusted in a Carlo Erba Elemental Analyser (Model 1108) and the released CO_2_ was analysed by MS. The δ^13^C=[(*R*_sample_–*R*_standard_)/*R*_standard_]×1000, where *R*_sample_ and *R*_standard_ are the ^13^C/^12^C ratio of the sample and standard (Pee Dee Belemnite), respectively. Photosynthetic carbon isotope discrimination based on leaf dry matter δ^13^C (∆_DM_) was calculated as described by Farquhar and Richards (1984):

$$\Delta_{DM}=\frac{\delta_{a}-\delta_{p}}{1+\delta_{p}}$$ (11)

where δ_a_ and δ_p_ are the δ^13^C values in the glasshouse air (assumed to be –8‰) and in the leaf bulk material, respectively.

### PWUE and PNUE calculations

Photosynthetic water use efficiency (PWUE) was calculated as *A* (µmol m^–2^ s^–1^)/*g*_s_ (mol m^–2^ s^–1^). Photosynthetic nitrogen use efficiency (PNUE) was calculated as *A* (µmol m^–2^ s^–1^)/leaf N_area_ (mmol m^–2^).

### Plant harvest

Plants were harvested 10–11 weeks after transplanting. At harvest, leaves were separated from stems. Total leaf area was determined using a Licor LI-3100A leaf area meter. Roots were washed free of soil. Plant materials were oven-dried at 80 ºC for 48 h before dry mass (DM) was measured. Total plant DM included leaf, stem, and root DM. Leaf nitrogen use efficiency (NUE) was calculated as the ratio of total plant DM (g per plant)/total leaf N content (mg).

### Statistical analysis

Growth, gas exchange, enzyme assay, and leaf nitrogen analyses were performed on 3–4 replicates per treatment combination (species×light). For species as a main effect, the relationship between various response variables and the main effect (species and light treatment) and their interactions were fitted using the linear model in R (R Foundation for Statistical Computing, Vienna, Austria) (R Core Team, 2015). Since the numbers of species within each subtype were unequal and measurements were taken on multiple individuals within a species, each unit cannot be considered as a true independent replicate. Therefore, a linear mixed effect model (lmer) was used to estimate the fixed effect associated with light treatment and subtype, where species were treated as a random variable. For each response variable, the models containing all possible fixed effects were fitted using the lme4 package (Bates *et al.*, 2013) in R. The model residues were tested for normality, and data transformation was carried out to achieve a normal distribution, if required. Significance tests were performed with a parametric bootstrap by using the ‘pbkrtest’ package in R (Halekoh and Højsgaard, 2014). Briefly, a linear mixed effect model (fitted with maximum likelihood), full and restricted models, was used as a sample to generate the likelihood ratio statistic (LRT) after 1000 bootstraps. To estimate the *P*-value, LRT divided by the number of degrees of freedom was assumed to be *F*-distributed where denominator degrees of freedom are determined by matching the first moment of reference distribution.

## Results

Throughout this study, the species effect was highly significant for all parameters and generally is not described below (Table 2).

### Plant growth and leaf chemistry

**Fig. 1.**
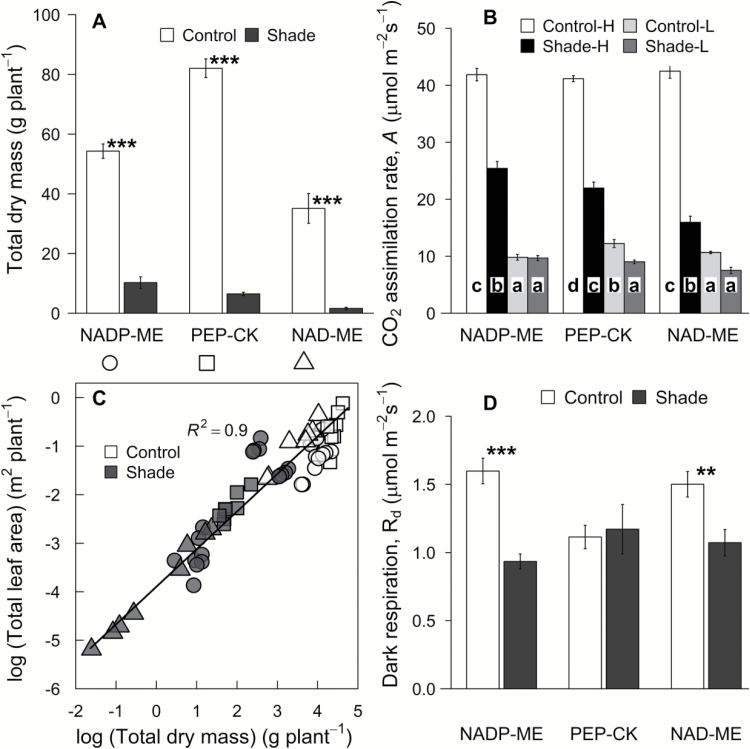
Total plant dry mass and leaf area. (A) Total plant dry mass (DM), (B) CO_2_ assimilation rate measured at ambient [CO_2_], (C) relationship between the log of total leaf area and the log of total DM, and (D) dark respiration (*R*_d_) for eight C_4_ grasses belonging to three biochemical subtypes and grown in control (full sunlight; white) or shade (16% of natural sunlight; black) environments. Each column represents the mean ±SE of subtype. For (A) and (D), statistical significance levels (*t*-test) for the growth condition within each subtype are shown: *P<0.05; **P<0.01; ***P<0.001. In (B), measurements were made at 2000 (HL) and 250 (LL) μmol quanta m^–2^ s^–1^ for both control and shade treatments. Letters indicate the ranking (from lowest=a) within each subtype using multiple-comparison Tukey’s post-hoc test.

Across treatments, PEP-CK species had a higher average plant DM and total leaf area relative to NADP-ME and NAD-ME species (*P*<0.05; Table 2; Fig. 1A; Supplementary Table S1). Shade reduced plant DM to a greater extent in NAD-ME (–95%) and PEP-CK (–92%) relative to NADP-ME (–81%) species (Table 2; Fig. 1A; Supplementary Table S1). Total plant DM and total leaf area were linearly correlated across the species and light treatments (*R*^2^=0.9 for the log relationship) (Fig. 1C). The effect of shading on plant DM and leaf area increased linearly with shaded plant DM (*R*^2^=0.97 and 0.89, respectively). The root to shoot ratio did not vary according to subtype but was substantially reduced by shade in all species (Table 2; Supplementary Table S1). There was no significant subtype effect on LMA, leaf N_mass_, or leaf N_area_, while shade reduced LMA and leaf N_area_ (but not leaf N_mass_) in most species (Table 2; Supplementary Table S1).

### Leaf gas exchange at low and high light

**Fig. 2.**
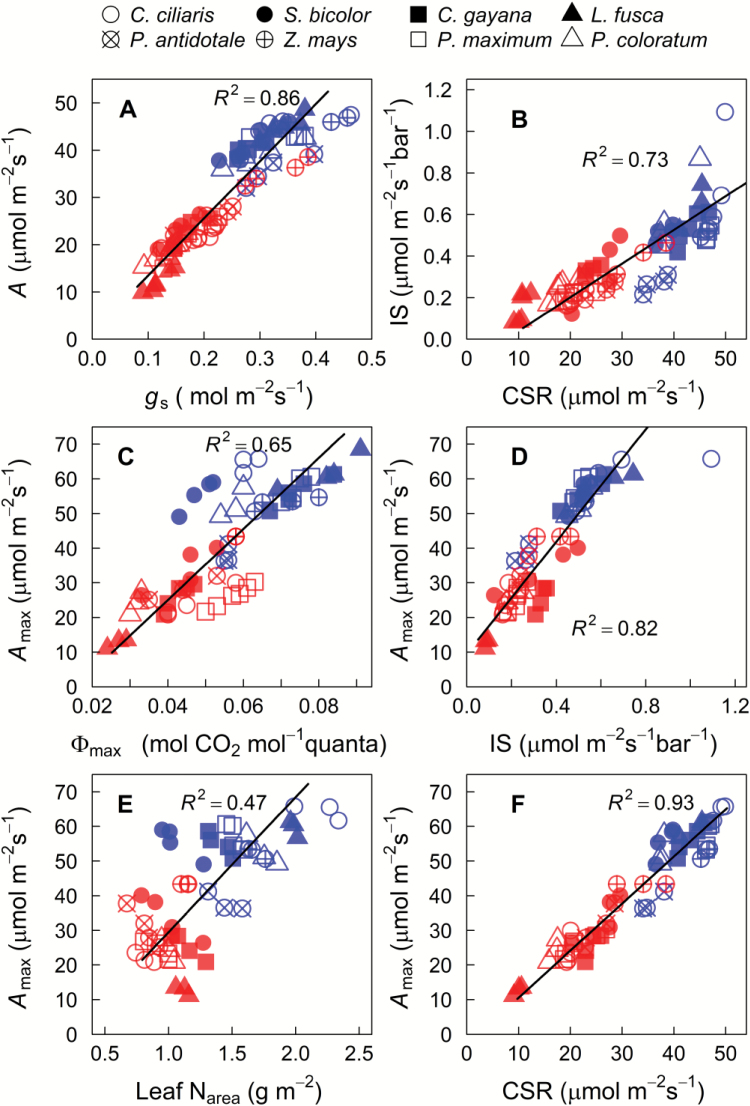
Relationships among physiological and *in vivo* derived parameters. CO_2_ assimilation (*A*_h_), stomatal conductance (*g*_h_), IS, and CSR derived from *A*–*C*_i_ curves measured at high light, *A*_max_ and Φ_max_ derived from light response curves measured at saturating [CO_2_], and leaf N_area_ for eight C_4_ grasses belonging to three biochemical subtypes grown in control (full sunlight; blue) or shade (16% of natural sunlight) (red) environments. Straight lines are linear regressions for all data points.

Overall, there was no significant subtype effect on CO_2_ assimilation rates and stomatal conductance measured at HL (*A*_h_ and *g*_sh_, respectively) and LL (*A*_l_ and *g*_sl_, respectively) (*P*>0.05, Table 2). However, there was a significant treatment and subtype×treatment effect on both *A*_h_ and *A*_l_ (*P*<0.05; Table 2). In particular, shade reduced *A*_h_ and *A*_l_ to a greater extent in NAD-ME (–62% and –27%, respectively) relative to PEP-CK (–46% and –15%, respectively) and NADP-ME –40% and 0%, respectively) species (Fig. 1B; Supplementary Table S2). Furthermore, under shade, NADP-ME species had the highest and NAD-ME species had the lowest *A*_h_ and *g*_h_, indicating that photosynthesis and stomatal conductance acclimated to shade more strongly in the latter species (Fig. 1B; Supplementary Table S2). The CO_2_ assimilation rate was strongly correlated with stomatal conductance across all species and treatments (*R*^2^=0.86) (Fig. 2A). Consequently, *C*_i_/*C*_a_ was constant and, together with PWUE, did not vary according to the subtype or light treatment (Table 2; Supplementary Table S2).

*R*_d_ did not differ between the subtypes but it was reduced by shade in NADP-ME and NAD-ME species (Fig. 1D; Table 2; Supplementary Table S2). For control plants, the *R*_d_/*A* ratio (measured at growth light) was lowest in PEP-CK species; shade increased *R*_d_/*A* less in NADP-ME (+158%) relative to PEP-CK (+374%) and NAD-ME (+341%) species (Table 2; Supplementary Table S2). CO_2_ assimilation rate measured at HL, *A*_h_, and *R*_d_ showed good linear relationships to leaf N_area_ across species (*R*^2^=0.59 and *R*^2^=0.54, respectively). PNUE was marginally lower (*P*=0.1) in NAD-ME relative to NADP-ME and PEP-CK species, and was reduced by shade mostly in NAD-ME (–28%) and PEP-CK (–19%) (Table 2; Supplementary Table S2). Leaf NUE was reduced to a greater extent in NADP-ME and NAD-ME species (–41% and –39%, respectively) relative to PEP-CK species (Table 2; Supplementary Table S1).

### Photosynthetic CO_2_ response curves

The initial slopes (ISs) of the *A*–*C*_i_ curvesand CO_2_-saturated rates (CSRs) were estimated from measurements at HL (Supplementary Fig. S2). In control plants, the IS and CSR did not vary with subtypes, but were reduced by shade to a greater extent in NAD-ME (–77% for IS and –64% for CSR) than PEP-CK (–49% for IS and CSR) and NADP-ME species (–46% for IS and –39% for CSR) (Tables 2, 3). Consequently, CSR of shaded plants was lowest in NAD-ME, intermediate in PEP-CK, and highest in NADP-ME species (Tables 2, 3). There was a strong linear relationship between IS and CSR irrespective of treatment and subtype (*R*^2^=0.73) (Fig. 2B).

### Photosynthetic light response curves

**Fig. 3.**
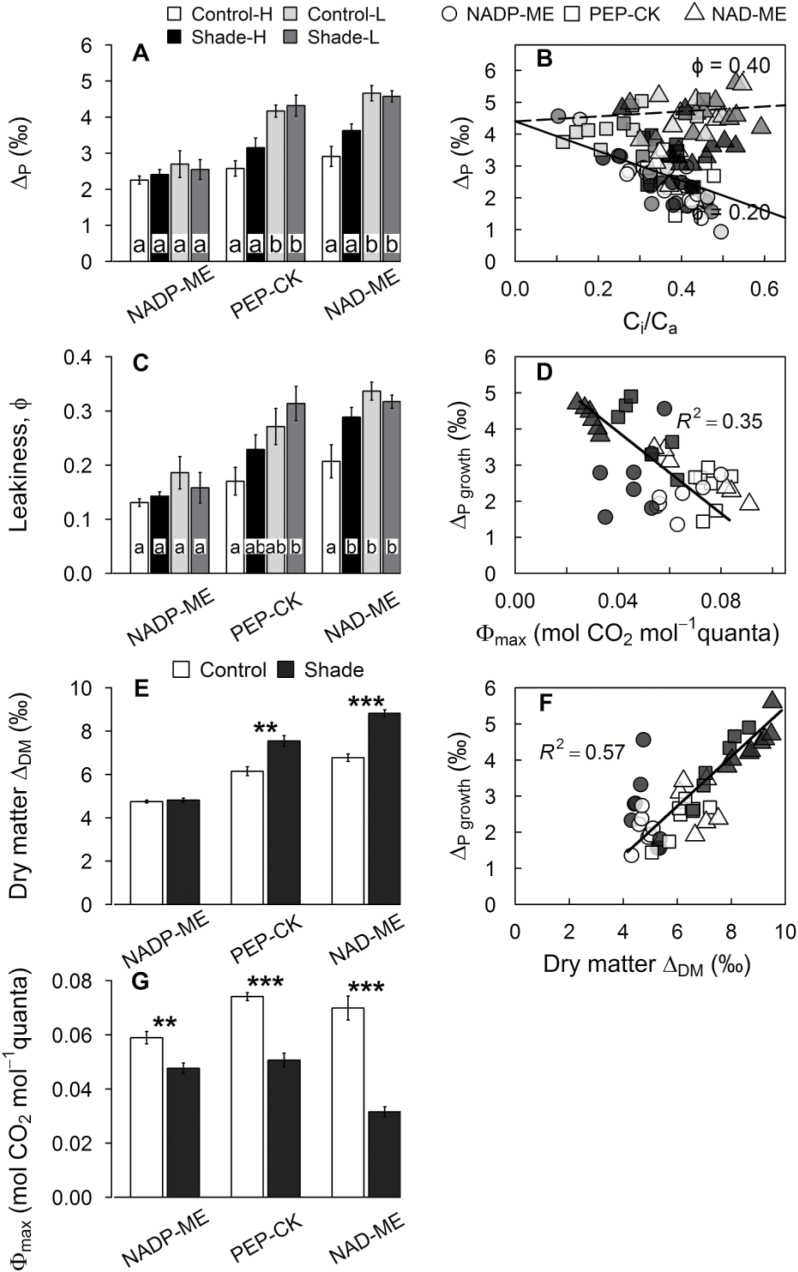
The CCM efficiency parameters. (A) Photosynthetic carbon isotope discrimination (∆_P_) measured concurrently with leaf gas exchange, (B) carbon isotope discrimination against the ratio of intercellular [CO_2_] to ambient [CO_2_] (*C*_i_/*C*_a_), (C) leakiness (ϕ) at measured light (h or l), (D) ∆_P_ measured at growth light (∆_growth_) against Ф_max_, (E) ∆_DM_ calculated from leaf dry matter δ^13^C, (F) ∆_P_ measured at growth light (∆_Pgrowth_) against ∆_DM_, and (G) and maximum quantum yield of PSII, Ф_max_, for C_4_ grasses belonging to three biochemical subtypes grown in control (full sunlight; white) or shade (16% of natural sunlight; black) environments. Each column represents the mean ±SE of subtype. Statistical significance levels (*t*-test) for the growth condition within each subtype are shown: *P<0.05; **P<0.01; ***P<0.001.

The light-saturated photosynthesis rate, *A*_max_, and photosynthetic quantum yield, Ф_max_, were estimated from the light response curves of photosynthesis measured at saturating CO_2_ (Supplementary Fig. S3). In control plants, *A*_max_ and Ф_max_ did not vary with subtypes (Tables 2, 3; Fig. 3G). Shade reduced *A*_max_ and Ф_max_ to a greater extent in NAD-ME species (–68% and –55%, respectively) than PEP-CK (–54% and –32%, respectively) and NADP-ME (–39% and –19%, respectively) species (Tables 2, 3). Consequently, Ф_max_ was lower in shaded NAD-ME species relative to their NADP-ME and PEP-CK counterparts (*P*<0.05), which indicates differential shade acclimation of photosynthetic capacity and quantum efficiency among the C_4_ subtypes. In control plants, the curvature (θ) was highest in NADP-ME (0.81) and lowest in NAD-ME species (0.53) (*P*<0.05) (Tables 2, 3). When all data were considered, *A*_max_ was well correlated with Ф_max_, IS, CSR, and leaf N_area_ (Fig. 2C–F).

### Carbon isotope discrimination

Concurrent measurements of ^13^CO_2_/^12^CO_2_ discrimination and leaf gas exchange showed that photosynthetic carbon isotope discrimination, ∆_P_, was independent of the C_4_ subtype at HL (Table 2). ∆_P_ tended to be lower in NADP-ME species relative to the other two subtypes, but differences were not significant, except when compared with the control plants measured at HL (Table 2; Supplementary Table S3). For NADP-ME species, ∆_P_ was unchanged by either LL or shade, while ∆_P_ increased in PEP-CK and NAD-ME species in response to both LL and shade (22–37%) (Fig. 3A; Supplementary Table S3).

Leakiness (ϕ) ranged between 0.15 and 0.35 across the C_4_ grasses and light treatments (Fig. 3B, C). For shaded plants measured at HL, NAD-ME species had higher leakiness (0.29) than NADP-ME species (0.14) (Supplemetary Table S3). Overall, NAD-ME species exhibited increased ϕ at LL and in the shade environment (Fig. 3C; Supplementary Table S3).

Photosynthetic carbon isotope discrimination derived from bulk leaf δ^13^C values, ∆_DM_, was significantly lower in NADP-ME species relative to the other two subtypes (*P*<0.05) (Table 2; Supplementary Table S3). Shade increased ∆_DM_ in NAD-ME and PEP-CK species only (+30% and +22%, respectively) (Fig. 3E; Table 2; Supplementary Table S3).

There was a strong linear relationship between photosynthetic discrimination measured at growth light, ∆_growth_, and ∆_DM_ (*R*^2^=0.56) (Fig. 3F). This relationship had an *x*-intercept of 0.9‰, which reflects the difference between ∆_growth_ and ∆_DM_ due to time-integrated changes in ambient ^13^CO_2_/^12^CO_2_ and post-photosynthetic fractionation. The good fit between ∆_growth_ and ∆_DM_ suggests that leaf δ^13^C is a good predictor of ∆_growth_, and perhaps *C*_i_/*C*_a_ (PWUE) for C_4_ grasses when changes are caused by a difference in light intensity. In addition, there was a significant, negative linear relationships between Ф_max_ and ∆_growth_ (*R*^2^=0.35) (Fig 3D).

### Activity of photosynthetic enzymes

**Fig. 4.**
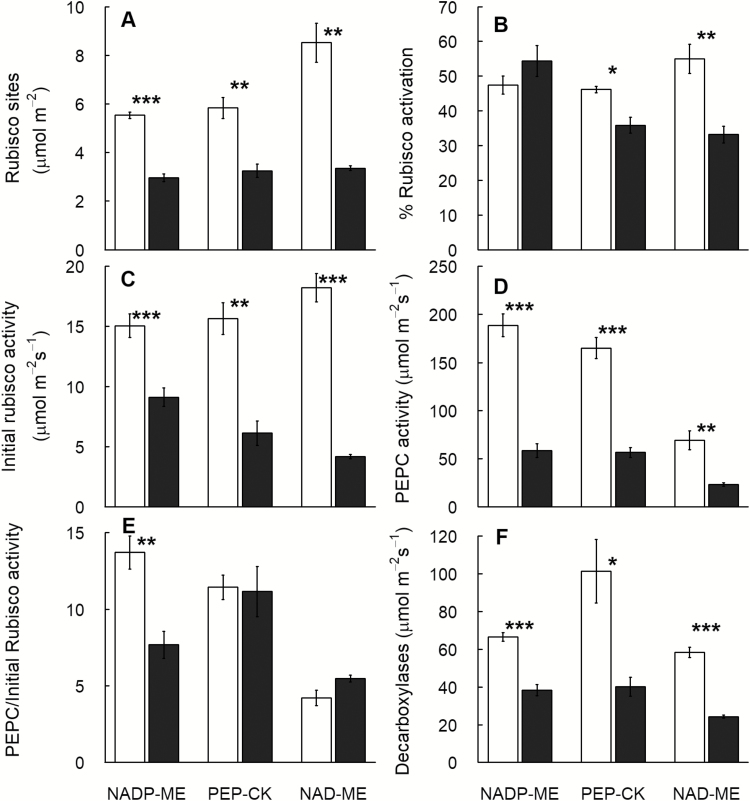
Shade acclimation of photosynthetic enzymes in C_4_ subtypes. (A) Rubisco sites, (B) % Rubisco activation, (C) Initial Rubisco activity, (D) PEPC activity, (E) PEPC to initial Rubisco activity ratio, and (F) decarboxylases activity for eight C_4_ grasses belonging to three biochemical subtypes and grown in control (full sunlight; white) or shade (16% of natural sunlight; black) environments. Each column represents the mean ±SE of subtype. Statistical significance levels (*t*-test) for the growth condition within each subtype are shown: *P<0.05; **P<0.01; ***P<0.001.

Control NAD-ME plants had the highest leaf content of Rubisco sites (*P*<0.05) (Fig. 4A; Table 2: Supplemetnary Table S4). Rubisco activation decreased significantly in NAD-ME (–40%) and PEP-CK (–22%) species, while it increased by ~15% in NADP-ME species (subtype×light *P*=0.07) (Fig. 4A, B; Table 2; Supplementary Table S4). Consequently, initial Rubisco activity did not differ according to the C_4_ subtype (*P*>0.05), and was reduced to a greater extent in NAD-ME relative to the PEP-CK and NADP-ME species under shade in all C_4_ grasses (subtype×treatment *P*<0.08) (Fig. 4C; Table 2; Supplementary Table S4). Soluble protein content decreased under shade by 12–67% depending on the species but not the subtype (Table 2; Supplementary Table S4).

In general, shade reduced PEPC activity by 49–84% in the C_4_ grasses. PEPC activity was higher in control NADP-ME plants and decreased to a lesser extent in NAD-ME (66%) than in NADP-ME (69%) and PEP-CK (65%) species (Fig. 4D; Table 2; Supplementary Table S4). Consequently, shade reduced the ratio of PEPC to initial Rubisco activity in NADP-ME (–44%) but not in PEP-CK (–3%) species, while this ratio tended to increase in NAD-ME species (+30%) (Fig. 4E; Supplementary Table S4,).

There was a significant relationship between the PEPC/initial Rubisco activity ratio and ∆_DM_ (*P*<0.05), and this relationship was stronger in shade (*R*^2^=0.49) than in control (*R*^2^=0.40) plants. In contrast, PEPC/initial Rubisco activity showed a weaker relationship to ∆_growth_ (*R*^2^=0.23) irrespective of treatment and subtype.

Activities of NADP-ME, PEP-CK, and NAD-ME enzymes were dominant in their respective subtype; however, substantial PEP-CK activity (6–25 µmol m^–2^ s^–1^) was measured in NADP-ME and NAD-ME species (Supplementary Table S4). Shade reduced the activity of NADP-ME, PEP-CK, and NAD-ME by 35–60, 52–64, and 49–57%, respectively (*P*<0.05). Shade also reduced the activity of total decarboxylases by 25–64% (*P*<0.05); the reduction was lower in NADP-ME (42%) relative to PEP-CK (60%) and NAD-ME (65%) species (Fig. 4F; Table 2; Supplementary Table S4).

**Fig. 5.**
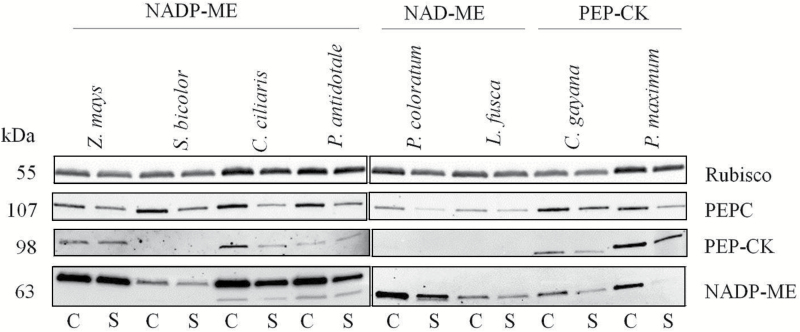
Immunoblot analysis of photosynthetic enzymes. Immunoblot analysis for the photosynthetic proteins Rubisco, PEPC, PEP-CK, and NADP-ME extracted from leaves of eight C_4_ grasses belonging to three biochemical subtypes in control (C) or shade (S) environments. Loaded volumes varied between 4 μl and 15 μl in order to normalize the protein content to a common leaf area. Because of the small gel size, a limited number of samples (8–9) was loaded on an individual gel. Finally, all immunoblots of the studied protein and species were arranged in a composite figure. For uniform visualization, gamma settings of individual images were adjusted. A protein ladder was used for individual immunoblots; for simplicity, band size is referred to numerically.

The detectability of enzyme activity was corroborated by immunodetection of the corresponding protein for all the photosynthetic enzymes assayed, except for NAD-ME where a suitable antibody was not available during this study (Fig. 5). Rubisco and PEPC proteins were detected in all species and treatments. Surprisingly, NADP-ME protein was detected in all C_4_ species, including NAD-ME and PEP-CK. This may be attributed to cross-reaction with a non-photosynthetic isomer of NADP-ME or NAD-ME proteins (Fig. 5). PEP-CK protein was strongly detectable in *Panicum maximum* and to a lesser extent in *Chloris gayana*, although *C. gayana* had the highest PEP-CK activity (Fig. 5; Supplementary Table S4). In addition, PEP-CK protein was detected in three NADP-ME species, but not in *Sorghum bicolor*, reflecting well the trends in PEP-CK activity (Fig. 5; Supplementary Table S4).

## Discussion

C_4_ photosynthesis is thought to be less plastic in response to shade than C_3_ photosynthesis due to its complex anatomy and biochemistry (Sage and McKown, 2006). Yet, some studies found similar photosynthetic responses to LL in C_3_ and C_4_ plants (Tazoe *et al.*, 2006, 2008; Pengelly *et al.*, 2010). These studies have focused on the response of selected C_4_ species to short-term changes in irradiance (Tazoe *et al.*, 2008; Pengelly *et al.*, 2010; Ubierna *et al.*, 2013; Bellasio and Griffiths, 2014*a*, *b*) or long-term adaptation to growth under LL (Kromdijk *et al.*, 2008, 2010; Tazoe *et al.*, 2008; Bellasio and Griffiths, 2014*b*). However, there is limited information about how LL responses differ across the C_4_ subtypes. Here, we present the first study comparing the short- and long-term responses to LL of C_4_ grasses with different biochemical subtypes. We hypothesized that (i) LL (short and long term) will compromise the CCM efficiency to a greater extent in NAD-ME than in PEP-CK or NADP-ME grasses due to a greater increase in leakiness (ϕ) in the former subtype; and (ii) shade will cause a greater photosynthetic acclimation in NAD-ME grasses than in NADP-ME and PEP-CK counterparts by virtue of their higher leaf N and Rubisco content. To evaluate these hypotheses, we grew eight C_4_ grasses belonging to three biochemical subtypes (NADP-ME, NAD-ME, and PEP-CK) under shade (16% sunlight) or control (full sunlight) conditions, and subsequently measured their photosynthetic characteristics under both LL and HL. Our results supported both hypotheses and demonstrated that LL compromised the CCM efficiency and photosynthetic quantum yield to a greater extent in NAD-ME relative to PEP-CK and NADP-ME species.

### Low light compromised the CCM efficiency most in NAD-ME followed by PEP-CK, but not in NADP-ME grasses

For the operational CCM, the C_4_ cycle must be faster than the C_3_ cycle (i.e. PEPC/initial Rubisco activity ratio >1) to concentrate CO_2_ inside the BSCs, some of which will inevitably leak back out to the MCs. In the short term, LL may compromise CCM efficiency by affecting the activity of the C_3_ cycle (e.g. Rubisco) more significantly than that of the C_4_ cycle (e.g. PEPC) and, hence, increasing ∆_P_, ϕ, and Ф_max_. Under LL, *A* is low and CO_2_ evolved during respiration can make an important contribution to the total CO_2_ concentration inside the BSCs. Larger *R*_d_/*A* ratios under LL can potentially lead to higher ∆_P_. Increased ∆_P_ due to respiratory CO_2_ does not involve an energy cost for the CCM but may lead to an overestimation of ϕ independently of Ф_max_. Long-term acclimation to LL may act to optimize CCM efficiency by reversing the negative short-term effects of LL (Bellasio and Griffiths, 2014*a*). Hence, we used the metrics PEPC/initial Rubisco activity, *R*_d_/*A*, ϕ, and Ф_max_ to evaluate the effects of LL during measurements and growth on the CCM efficiency of the various C_4_ subtypes. Consequently, increased ϕ, PEPC/initial Rubisco activity, and *R*_d_/*A* can be interpreted as a less efficient photosynthetic process. Assuming that bundle sheath conductance does not change with irradiance, increased ∆_P_ and ϕ can be interpreted as a less efficient CCM and an indication of imbalance between the C_3_ and C_4_ cycles (von Caemmerer, 2000).

#### Response of NADP-ME grasses to LL

In the NADP-ME grasses, ∆_P_, ∆_DM_, and ϕ were not significantly impacted by LL during either measurement or growth (Fig. 3A, C, E), suggesting that in this subtype, the co-ordination between the C_3_ and C_4_ cycles was largely maintained despite changes in the light environment. Our results are in agreement with previous reports that ∆_P_ was insensitive to LL during the measurements for NADP-ME species (Henderson *et al.*, 1992; Kubásek *et al.*, 2007; Cousins *et al.*, 2008) as well as ∆_DM_ being insensitive to growth under shade in NADP-ME *Zea mays* (Sharwood *et al.*, 2014). NADP-ME species grown under shade had lower PEPC/initial Rubisco activity (–44%) and higher *R*_d_/*A* (+158% at growth light) than the control plants. Decreased PEPC/initial Rubisco activity is expected to reduce ∆_P_, ∆_DM_, and ϕ, while increased *R*_d_/*A* will have the opposite effect under shade. The combined opposing effects may explain the insensitivity of ∆_P_, ∆_DM_, and ϕ in response to the shade environment. In line with this conclusion, NADP-ME species showed the lowest reduction in Ф_max_ (–19%), and it is likely that the contribution from photorespiration was negligible in the low-O_2_-evolving BSC chloroplasts of NADP-ME species (Ghannoum *et al.*, 2005). Previous work describing the shade acclimation of the NADP-ME of *Z. mays* suggested that this species reduced the ATP cost of the CCM under shade by reducing the PEPC activity more than the C_3_ cycle activity, which resulted in low ϕ values (Bellasio and Griffiths, 2014*a*). Our findings support this argument by providing direct measurements of *in vitro* C_4_ and C_3_ cycle enzymes as well as Ф_max_. This argument is also supported by the modelling approach of Wang *et al.* (2014) who suggested that the NADP-ME biochemical pathway is favoured at LL. In contrast to our results, differential responses of C_4_ and C_3_ cycle enzymes were reported in earlier studies with NADP-ME species (Sugiyama *et al.*, 1984; Ward and Woolhouse, 1986*a*; Sharwood *et al.*, 2014). This discrepancy may be related to differences in the intensity of the shade treatment used. In addition, these studies considered total (rather than initial) Rubisco activity for calculating the PEPC/Rubisco activity ratio.

It is worth noting that, in addition to NADP-ME decarboxylase activity, *Z. mays* and *Cenchrus ciliaris* showed significant activity of PEP-CK decarboxylase (16–25 µmol m^–2^ s^–1^), while *S. bicolor* and *Panicum antidotale* appeared as true NADP-ME types. Without considering cytosolic resistance of BSCs to CO_2_, Wang *et al.* (2014) suggested the possibility of higher leakiness in the C_4_ photosynthesis model with mixed decarboxylase pathways. This was not validated in our study. Bellasio and Griffiths (2014*c*) also suggested that the engagement of the secondary PEP-CK pathway in an NADP-ME species enables the CCM to regulate an optimal BSC [CO_2_] under changing light conditions. These predictions were indirectly validated in our study. Under LL, *Z. mays* showed higher ∆_P_ and ϕ but similar Ф_max_ relative to the other two NADP-ME species, *P. antidotale* and *S. bicolor* (Supplementary Table S4). However, the link between ∆_P_, CCM efficiency, and PEP-CK activity as a secondary decarboxylase in NADP-ME species requires further investigation.

#### Response of PEP-CK and NAD-ME grasses to LL

PEP-CK and NAD-ME plants had larger ∆_DM_ under shade relative to the control condition (Fig. 3E). Previous studies have shown similar ∆_DM_ responses to shade across the C_4_ subtypes (Buchmann *et al.*, 1996; Tazoe *et al.*, 2006). In PEP-CK and NAD-ME grasses, instantaneous measurements of ∆_P_ and ϕ increased at LL, and the difference between the light treatments was highly significant when the comparison was made between control and shade plants measured under their respective growth irradiance (Fig. 3A, C). These results indicate that LL resulted in a less efficient CCM in PEP-CK and NAD-ME grasses. Additionally, the relative increase in ϕ from HL to LL was larger for control (+60%) than shade (+12%) plants. This is in line with Tazoe *et al.* (2008) and suggests that a degree of acclimation to the shade condition mitigated the negative effects of LL observed in response to short-term light changes during measurements. Our results with PEP-CK and NAD-ME grasses are in agreement with previous reports of increased ∆_P_ and ϕ with short-term exposure to LL (Henderson *et al.*, 1992; Cousins *et al.*, 2008).

In NAD-ME species, shade increased the PEPC/initial Rubisco activity (+30%) and *R*_d_/*A* (+341% at growth light) and decreased Ф_max_ (–55%). Species of the NAD-ME subtype possess significant PSII activity in the BSC (Ghannoum *et al.*, 2005), and hence potentially high [O_2_]. PEP-CK species exhibited intermediate responses to shade relative to the other two subtypes. In PEP-CK species, the PEPC/initial Rubisco activity ratio was not affected by shade, but the *R*_d_/*A* ratio was larger (+374%) in shade than in control plants (Supplementary Table S2). Further, the reduction of Ф_max_ under shade was intermediate in PEP-CK species (–32%) relative to NADP-ME (–19%) and NAD-ME (–55%). We also have evidence that PEP-CK species possess significant PSII activity in BSCs (Pinto, 2015). Accordingly, ∆_P_, ∆_DM_, and ϕ increased under shade in PEP-CK species to a similar extent relative to NAD-ME counterparts (Fig. 3A, C, E). Thus, in line with the first hypothesis, our results demonstrated that LL compromised the CCM efficiency of NAD-ME species more than that of PEP-CK species, while CCM efficiency was largely maintained in NADP-ME species under LL.

### Shade induced larger photosynthetic down-regulation in NAD-ME relative to NADP-ME and PEP-CK species

In the current study, shade down-regulated *A*_h_ and light-saturated photosynthesis, *A*_max_, in NAD-ME (–68%) to a greater extent than in PEP-CK (–54%) and NADP-ME (–39%) species, indicating stronger photosynthetic acclimation to shade in the former subtype (Table 3). Shade equally reduced PEPC activity in all the C_4_ subtypes (–68%), while Rubisco sites and activation were more profoundly reduced in NAD-ME (–60% and –40%, respectively) relative to the PEP-CK (–48% and –22%, respectively) and NADP-ME (–47% and +15%, respectively) species (Supplementary Table S4). Similar large reductions in Rubisco content and activity (>55%) were reported in studies using C_3_ species (Evans, 1988). In shade-grown C_4_ species, inconsistent changes in Rubisco content and activity have been observed (Winter *et al.*, 1982; Ward and Woolhouse, 1986*a*, *b*; Tazoe *et al.*, 2006), with an average reduction of 29% (Sage and McKown, 2006). A relevant study by Ward and Woolhouse (1986*a*) subjecting C_4_-NADP-ME grasses to deep shade reported a greater reduction in Rubisco activity in species from the open habitat (34%) relative to the shade habitat (3%). Likewise, NAD-ME species generally originate from relatively more open habitats than NADP-ME and PEP-CK species (Vogel *et al.*, 1986; Hattersley, 1992; Schulze *et al.*, 1996; Liu *et al.*, 2012). In addition, similar to the C_3_ species, NAD-ME grasses may have a greater N flexibility by having higher leaf N relative to NADP-ME and PEP-CK counterparts.

**Table 3.** Parameters derived from *A*–*C*_i_ and light response curves for eight C_4_ grasses grown under control (full sunlight) or shade (16% of natural sunlight) environments

Parameter	Treatment	Subtype			C_4_
		NADP-ME	PEP-CK	NAD-ME	
IS at HL (µmol m^–2^ s^–1^ bar^–1^)	Control	0.5 ± 0.06 a	0.52 ± 0.02 a	0.62 ± 0.06 a	0.53 ± 0.03
	Shade	0.27 ± 0.03 a	0.27 ± 0.02 a	0.2 ± 0.02 a	0.25 ± 0.02
	% change	**–46**	**–49**	**–77**	**–54**
CSR at HL (µmol m^–2^ s^–1^)	Control	42 ± 1 a	45 ± 1 a	41 ± 1 a	43 ± 1
	Shade	25 ± 1 b	23 ± 1 ab	15 ± 1 a	21 ± 1
	% change	**–39**	**–49**	**–64**	**–50**
IS/CSR at HL (×10^3^)	Control	12 ± 1 a	12 ± 0 a	15 ± 1 a	12 ± 1
	Shade	10 ± 1 a	12 ± 1 a	13 ± 1 a	12 ± 1
	% change	–12	1	–9	–6
*A*_max_ (µmol m^–2^ s^–1^)	Control	53 ± 3 a	57 ± 1 a	58 ± 2 a	55 ± 1
	Shade	32 ± 2 a	26 ± 1 a	18 ± 3 a	28 ± 1
	% change	**–39**	**–54**	**–68**	**50**
Maximum quantum yield (Ф_max_) (mol mol^–1^)	Control	0.06 ± 0 a	0.07 ± 0 a	0.07 ± 0 a	0.07 ± 0
	Shade	0.05 ± 0 b	0.05 ± 0 b	0.03 ± 0 a	0.05 ± 0
	% change	**–19**	**–32**	**–55**	**–32**
Curvature (θ) of light response curve	Control	0.81 ± 0.04 b	0.57 ± 0.03 ab	0.53 ± 0.04 a	0.66 ± 0.03
	Shade	0.61 ± 0.04 a	0.64 ± 0.05 a	0.64 ± 0.05 a	0.62 ± 0.03
	% change	**–25**	12	19	–6

Values are means ±SE (*n*=3–4). The ranking (from lowest=a) of subtypes within each single row using multiple-comparison Tukey’s post-hoc test. Values followed by the same letter are not significantly different at the 5% level. Significant fold changes are shown in bold (*P*<0.05).

Reduced Rubisco content is a common photosynthetic acclimation response to shade allowing optimal nitrogen allocation for maximal light harvesting (Boardman, 1977; Hikosaka and Terashima, 1995; Evans and Poorter, 2001; Walters, 2005). Consequently, lower Rubisco activity and activation may indicate a shift in photosynthetic limitation from Rubisco (sun leaves) to electron transport (shade leaves) (Evans, 1988; Evans and Poorter, 2001). Under shade, NAD-ME species had lower Ф_max_ relative to NADP-ME and PEP-CK counterparts (Fig. 3). This difference may be attributed to a greater inefficiency of the NAD-ME CCM, especially under shade as argued above. It could also be attributed to inherent inefficiencies of the light conversion apparatus in the NAD-ME subtype due to the burden of operating two fully fledged linear electron transport systems with granal chloroplasts in both MCs and BSCs. This is not the case for the NADP-ME subtype, and somewhat intermediate for the PEP-CK subtype (Ghannoum *et al.*, 2005, 2011). Taken together, these findings support our second hypothesis stating that larger photosynthetic reduction and acclimation in NAD-ME species is associated with their lower Rubisco activation and lower quantum efficiency relative to PEP-CK and NADP-ME species.

### Whole-plant implications of differential shade acclimation among the C_4_ subtypes

In the current study, CO_2_ assimilation at growth light was equally reduced in NAD-ME (–81%), PEP-CK (–79%), and NADP-ME (–76%) species. However, total leaf area and plant DM were more profoundly reduced in NAD-ME (–92% and –95%, respectively) than in PEP-CK (–81% and –98%, respectively) and NADP-ME (–73% and –81%, respectively) species. In addition to reduced leaf area, the larger reduction in plant DM for NAD-ME and PEP-CK species could also be attributed to reduced CCM efficiency and Ф_max_. Further, the *R*_d_/*A* ratio increased to a greater extent in NAD-ME and PEP-CK (~3.5-fold) than in NADP-ME species (1.6-fold) under shade, which may result in a greater C loss in these two subtypes. Taken together, these findings suggest that shade may favour NADP-ME species due to their efficient CCM and quantum yield, and lower photosynthetic down-regulation and lower increase in *R*_d_/*A* relative to NAD-ME and PEP-CK species.

This conclusion is supported by ecological observations. NAD-ME species are preferentially found in open and arid habitats relative to the other two C_4_ subtypes (Osmond *et al.*, 1982; Vogel *et al.*, 1986; Hattersley, 1992; Schulze *et al.*, 1996; Liu *et al.*, 2012). On the one hand, most of the understorey C_4_ grasses belong to the NADP-ME subtypes, such as *Setaria* species (Schulze *et al.*, 1996), *Paspalum* species (Ward and Woolhouse, 1986*b*; Klink and Joly, 1989; Firth *et al.*, 2002), and *Microstegium vimineum* (Barden, 1987). Moreover, NADP-ME species form dense canopy crops such as maize, Sorghum, Miscanthus, and sugarcane where most leaves are shaded (Sage, 2014).

It should be noted that a different light spectrum affects CCM efficiency in C_4_ photosynthesis (Sun *et al.*, 2012). The light spectrum may vary in natural shade settings due to growth season, canopy compositions, and architecture (Ross *et al.*, 1986; Messier *et al.*, 1998). Therefore, an interaction effect between subtype and light spectral composition cannot be ignored, and further investigations are warranted.

### Conclusion

Using C_4_ grass from three biochemical subtypes and grown under full sunlight and shade (16% of full sunlight) conditions equivalent to the light environment prevailing in lower crop canopies or forest understorey, this study demonstrated that NAD-ME and to a lesser extent PEP-CK species were generally outperformed by NADP-ME species under shade. This response was underpinned by a more efficient CCM and quantum yield in NADP-ME. These findings were corroborated by *in vivo* and *in vitro* measurements of C_3_ and C_4_ cycle enzymes, maximum quantum yield of PSII (Ф_max_), photosynthetic carbon isotope discrimination (Δ_P_), leaf dry matter δ^13^C, and total plant dry mass. Future research is needed to quantify the impact of respiration and photorespiration on carbon isotope discrimination (∆_P_) in the three biochemical subtypes of C_4_ photosynthesis, as well as the significance of the secondary PEP-CK decarboxylase on photosynthetic responses to shade.

## Supplementary data

Supplementary data are available at *JXB* online.

Table S1. Summary of plant growth parameters.

Table S2. Summary of gas exchange parameters.

Table S3. Summary of carbon isotope discrimination parameters.

Table S4. Summary of biochemical parameters.

Table S5. Definitions and units for variables described in the text.

Fig. S1. Glasshouse growth conditions.

Fig. S2. Photosynthetic CO_2_ response curves (*A*–*C*_i_) of C_4_ grasses.

Fig. S3. Photosynthetic light response curves for C_4_ grasses.

Fig. S4. Sensitivity of leakiness at low light

Appendix S1. Leakiness calculations at low light

Supplementary Tables S1-S5

Supplementary Figures S1-S4

Appendix S1
