# Supplementary material for: Shade compromises the photosynthetic efficiency of NADP-ME less than that of PEP-CK and NAD-ME C4 grasses
Source: J Exp Bot. 2018 Apr 5;69(12):3053–68. doi: 10.1093/jxb/ery129 (PMC5972597; doi:10.1093/jxb/ery129)
Supplement: Supplementary Tables S1-S5 [file ery129_suppl_supplementary_tables_s1-s5.pdf]

Table S1. Summary of plant growth parameters.
Growth parameters of eight C_4 grasses grown in control (full sunlight) or shade (16% of natural sunlight) environments. Values are means ± SE (n = 3-4). The ranking (from lowest = a) of subtypes within each single row using multiple-comparison Tukey's *post hoc* test. Values followed by the same letter are not significantly different at the 5% level. Significant percent changes are shown in bold ($p < 0.05$).

**Parameter**	**Treatment**	**Subtype**			**C_4**
		**NADP-ME**	**PEP-CK**	**NAD-ME**	
Total DM (g plant^{-1})	Control	54.3±2.7ab	82.1±3.7b	35.1±5.9a	57.2±3.8
	Shade	10.2±2.2a	6.5±0.7a	1.6±0.5a	7.1±1.3
	% change	**-81**	**-92**	**-95**	**-88**
Total leaf area (m^2 plant^{-1})	Control	0.35±0.03a	0.56±0.07a	0.36±0.08a	0.41±0.03
	Shade	0.16±0.04a	0.11±0.01a	0.03±0.01a	0.12±0.02
	% change	**-53**	**-81**	**-92**	**0.29**
Root/shoot DM	Control	0.42±0.04a	0.32±0.02a	0.37±0.06a	0.38±0.03
	Shade	0.2±0.01b	0.12±0a	0.19±0.02ab	0.17±0.01
	% change	**-53**	**-62**	**-49**	**-54**
LMA(g m^{-2})	Control	41±3a	34±1a	37±2a	38±2
	Shade	25±2a	27±2a	22±1a	25±1
	% change	**-39**	**-20**	**-39**	**-35**
Leaf N_{mass} (mg g^{-1})	Control	38±1a	43±1ab	48±2b	42±1
	Shade	39±2a	39±2a	46±1a	41±1
	% change	2	-10	-4	-3
Leaf N_{area} (g m^{-2})	Control	1.56±0.13a	1.46±0.03a	1.81±0.08a	1.59±0.07
	Shade	0.94±0.05a	1.03±0.05a	1.02±0.03a	0.99±0.03
	% change	**-39**	**-29**	**-43**	**-38**
Plant NUE	Control	120±5b	86±7ab	72±6a	99±5
	Shade	71±4b	60±1ab	43±4a	61±3
	% change	**-41**	**-31**	**-39**	**-38**

Table S2. Summary of gas exchange parameters.
Gas exchange parameters of eight C_4 grasses grown in control (full sunlight) or shade (16% of natural sunlight) environments. Values are means ± SE (n = 3-4). The ranking (from lowest = a) of subtypes within each single row using multiple-comparison Tukey's *post hoc* test. Values followed by the same letter are not significantly different at the 5% level. Significant percent changes are shown in bold ($p < 0.05$).

**Parameter**	**Treatment**	**Subtype**			**C_4**
		**NADP-ME**	**PEP-CK**	**NAD-ME**	
CO_2 assimilation at HL, A_h (μmol $m^{-2}s^{-1}$)	Control	42±1a	41±1a	42±1a	42±1
	Shade	25±1b	22±1ab	16±1a	22±1
	% change	**-39**	**-47**	**-62**	**-48**
CO_2 assimilation at LL, A_l (μmol $m^{-2}s^{-1}$)	Control	10±1a	12±1a	11±0a	11±0
	Shade	10±0a	9±0a	8±1a	9±0
	% change	0	**-26**	**-30**	**-16**
Conductnace at HL, $g_{s\,h}$ (μmol $m^{-2}s^{-1}$)	Control	0.33±0.02a	0.31±0.01a	0.33±0.01a	0.32±0.01
	Shade	0.21±0.02b	0.17±0.01ab	0.13±0.01a	0.18±0.01
	% change	**-35**	**-43**	**-60**	**-45**
Conductnace at LL, $g_{s\,l}$ (μmol $m^{-2}s^{-1}$)	Control	0.07±0a	0.07±0a	0.07±0.01a	0.07±0
	Shade	0.07±0.01a	0.05±0a	0.06±0.01a	0.06±0
	% change	5	**-28**	-14	-10
Dark respiration, R_d (μmol m^{-2} s^{-1})	Control	1.6±0.1b	1.1±0.1a	1.5±0.1ab	1.4±0.1
	Shade	0.9±0.1a	1.2±0.2a	1.1±0.1a	1.1±0.1
	% change	**-42**	5	**-29**	**-25**
R_d/A_{GROWTH} (x 100)	Control	3.7±0.2b	2.7±0.2a	3.5±0.2ab	3.3±0.1
	Shade	9.7±0.6a	12.8±1.9a	15.7±2.2a	12.3±1
	% change	**158**	**373**	**341**	**269**
PWUE (μmol CO_2 mol^{-1} H_2O)	Control	130±5a	137±5a	132±5a	133±3
	Shade	121±5a	128±5a	124±6a	124±3
	% change	-7	-6	-6	**-7**
PNUE (μmol CO_2 s^{-1} g^{-1} N)	Control	29±2a	29±0a	23±0a	28±1
	Shade	29±1b	23±1ab	17±2a	24±1
	% change	0	**-19**	**-28**	**-12**

Table S3. Summary of carbon isotope discrimination parameters.
Carbon isotope discrimination parameters for eight C_4 grasses grown at control (full sunlight) or shade (16% of natural sunlight) environments. Values are means ± SE (n = 3-4). The ranking (from lowest = a) of subtypes within each single row using multiple-comparison Tukey's *post hoc* test. Values followed by the same letter are not significantly different at the 5% level. Significant percent changes are shown in bold ($p < 0.05$).

**Parameter**	**Treatment**	**Subtype**			**C_4**
		**NADP-ME**	**PEP-CK**	**NAD-ME**	
C_i/C_a at HL	Control	0.32±0.02a	0.3±0.02a	0.36±0.02a	0.32±0.01
	Shade	0.4±0.02a	0.4±0.02a	0.43±0.03a	0.41±0.01
	% change	**28**	**33**	19	**27**
C_i/C_a at LL	Control	0.35±0.02a	0.26±0.04a	0.37±0.08a	0.33±0.02
	Shade	0.35±0.04a	0.24±0.02a	0.44±0.04a	0.34±0.02
	% change	0	-7	19	4
Δ_P (‰) at HL	Control	2.26±0.17a	2.58±0.21a	2.91±0.28a	2.57±0.13
	Shade	2.41±0.17a	3.15±0.29a	3.63±0.19a	2.98±0.17
	% change	7	22	**25**	16
Δ_P (‰) at LL	Control	2.7±0.48a	4.17±0.17a	4.66±0.21a	3.95±0.23
	Shade	2.45±0.3a	4.07±0.35a	4.58±0.15a	3.69±0.23
	% change	-9	-2	-2	-3
Leakiness (ϕ_l) at HL	Control	0.13±0.01a	0.17±0.03a	0.21±0.03a	0.17±0.01
	Shade	0.14±0.01a	0.23±0.03ab	0.29±0.02b	0.22±0.02
	% change	9	34	**39**	**27**
Leakiness (ϕ_h) at LL	Control	0.19±0.04a	0.27±0.03a	0.34±0.02a	0.27±0.02
	Shade	0.14±0.03a	0.26±0.06a	0.32±0.01a	0.24±0.02
	% change	-26	-1	-5	-3
Dry matter photosynthetic C-isotope discrimination, Δ_{DM} (‰)	Control	4.8±0.1a	6.1±0.3b	6.8±0.2b	5.5±0.2
	Shade	5.2±0.2a	7.5±0.3b	8.8±0.2b	6.7±0.3
	% change	**9**	**22**	**30**	**21**

Table S4. Summary of biochemical parameters.

Biochemical parameters of eight C_4 grasses grown at control or shade (16% of natural sunlight) environments. Values are means ± SE (n = 3-4). The ranking (from lowest = a) of subtypes within each single row using multiple-comparison Tukey's *post hoc* test. Values followed by the same letter are not significantly different at the 5% level. Significant percent changes are shown in bold (p < 0.05).

Parameater	Treat	NADP-ME				PEP-CK		NAD-ME		Subtype			Total
		P. antidotale	*C. ciliaris*	*S. bicolor*	*Z. mays*	*M. maximus*	*C. gayana*	*P. coloratum*	*L. fusca*	NADP-ME	PEP-CK	NAD-ME	C_4
Rubisco activity	Control	33±1a	31±1a	29±1a	31±2a	29±4a	36±4a	37±3a	29±1a	31±1a	32±3a	33±2a	32
($\mu mol\ m^{-2}s^{-1}$)	Shade	16±1ab	13±1a	16±2ab	21±2b	11±2a	23±2b	11±1a	15±1ab	16±1a	17±3a	13±1a	16
	% change	**-50**	**-56**	**-45**	**-34**	-63	**-37**	**-71**	**-47**	**-47**	**-48**	**-60**	**-51**
Rubisco sites	Control	6.2±0.1a	5.1±0.2a	5±0.2a	5.7±0.3a	5.5±0.8a	6.3±0.8a	11±1b	6.7±0.2a	5.5±0.2a	5.8±0.5ab	8.5±1b	6.3±0.4
($\mu mol\ m^{-2}$)	Shade	2.9±0.2ac	2.2±0.1a	3.1±0.4ac	3.8±0.4bc	2.5±0.2ab	4±0.4c	3.2±0.2ac	3.5±0.2ac	3±0.2a	3.2±0.4a	3.4±0.2a	3.1±0.1
	% change	**-52**	**-56**	**-39**	**-34**	**-54**	-37	**-71**	**-47**	**-47**	**-44**	**-61**	**-51**
Rubisco	Control	42±4a	41±6a	47±5ab	62±1ab	44±2ab	48±1ab	45±1ab	65±7b	47±3a	46±1a	55±5a	49±2
activation (%)	Shade	32±6ab	83±5d	44±1bc	58±1cd	36±5ab	36±5ab	41±1bc	25±1a	54±6a	36±3a	33±4a	44±4
	% change	-24	**102**	-6	**-7**	-20	-25	-8	**-62**	15	**-22**	**-40**	-9
PEPC activity	Control	184±4d	128±6bc	274±13e	193±4d	169±22cd	160±16cd	94±12ab	51±11a	189±13b	165±13ab	69±12a	154±12
($\mu mol\ m^{-2}s^{-1}$)	Shade	46±8bc	20±2a	77±8cd	98±6d	48±6bc	65±11cd	27±3ab	19±3a	59±9a	57±7a	23±3a	50±6
	% change	**-75**	**-84**	**-72**	**-49**	**-72**	**-59**	**-71**	-62	**-69**	**-66**	**-66**	**-68**
PEPC/Initial	Control	14±1.6c	10.9±1.9bc	20.1±1.8d	9.6±0.5bc	13.6±1cd	9.3±0.5ac	5.6±0.5ab	2.9±0.3a	13.7±1.3b	11.4±1.1at	4.2±0.7a	11±1
Rubisco activity	Shade	8.9±1.1ac	1.8±0.2a	11.8±1.4bc	8.2±0.5ac	14.2±3.8c	8.1±0.5ac	6±0.5ab	5±0.3ab	7.7±1.2a	11.2±2.2a	5.5±0.3a	8±0.9
	% change	**-36.18**	**-83.65**	**-41.22**	-14.7	4.84	-13.15	7.45	**73.32**	**-43.93**	-2.48	29.79	**-27.22**
NADP-ME	Control	57±2c	47±2b	50±2bc	49±3b	1±0a	1±0a	4±0a	3±1a	51±1b	1±0a	3±0a	28±5
activity	Shade	23±2b	25±1bc	33±1c	43±3d	0±0a	1±0a	1±0a	1±0a	31±2b	1±0a	1±0a	16±3
($\mu mol\ m^{-2}s^{-1}$)	% change	**-60**	**-46**	**-35**	-13	**-77**	2	**-74**	**-67**	**-40**	-43	**-70**	**-100**
NAD-ME	Control	3±0a	1±0a	2±0a	3±0a	3±1a	3±0a	35±1b	36±3b	2±0a	3±0a	35±2b	10±3
activity	Shade	1±0a	1±0a	1±0a	2±0a	1±0a	2±0a	15±1b	18±1b	1±0a	1±0a	17±1b	5±1
($\mu mol\ m^{-2}s^{-1}$)	% change	**-56**	-26	-45	-8	-65	-44	**-57**	**-49**	**-38**	**-56**	**-53**	-50
PEP-CK	Control	6±1ab	16±1bc	2±0a	25±1c	46±3d	145±4e	20±3c	20±3c	11±3a	103±20b	20±2a	37±9
activity	Shade	3±1a	5±1ab	0±0a	13±1b	22±2c	52±3d	9±0ab	5±0a	6±2a	35±6b	7±1a	14±3
($\mu mol\ m^{-2}s^{-1}$)	% change	**-45**	**-68**	**-78**	**-47**	**-52**	**-64**	-55	**-76**	-50	**-66**	**-66**	**-62**
Protein ($g\ m^{-2}$)	Control	5.1±0.2ab	4.8±0.4ab	4.2±0.5ab	5.2±0.3ab	5.3±0.7ab	3.5±0.3a	5.8±0.5b	4.4±0.5ab	4.8±0.2a	4.4±0.5a	5±0.4a	4.8±0.2
	Shade	2.3±0.4ab	1.7±0.1a	3.7±0.3b	3.3±0.1b	1.8±0.2a	2.5±0.3ab	2.2±0.1ab	2.8±0.5ab	2.8±0.3a	2.2±0.2a	2.5±0.3a	2.5±0.2
	% change	**-54**	**-64**	-12	**-37**	**-67**	-27	**-62**	-37	**-42**	**-51**	**-50**	**-47**

Table S5. Definitions and units for variable described in the text.

Variable	Definition	Values/Calculation/Units
A	photosynthetic rate	µmol m^{-2} s^{-1}
g_{bs}	bundle-sheath conductance to CO_2 (von Caemmerer, 2000)	g_{bs} = 0.003 mol m^{-2} s^{-1}
ϕ_h	leakiness at high light (von Caemmerer et al., 2014)	unitless
ϕ_l	leakiness at low light (Ubierna et al., 2013)	unitless
a_b	^{13}C fractionation across the boundary layer	2.9 ‰
a	^{13}C fractionation due to diffusion of CO_2 in air	4.4 ‰
a'	weighted fractionation across the boundary layer and stomata in series	$a' = \frac{a_b(C_a - C_{ls}) + a(C_{ls} - C_i)}{C_a - C_i}$
a_i	fractionation factor associated with the dissolution of CO_2 and diffusion through water	$a_i = s$ = 1.8 ‰
s	fractionation during leakage of CO_2 out of the bundle-sheath cells (Henderson et al. 1992)	1.8 ‰
f	fractionation associated with photorespiration	11.6 ‰
t	ternary effect of transpiration rate on the carbon isotope discrimination during CO_2 assimilation	$t = \frac{(1+a')\cdot E}{2\cdot g_{ac}^{t}}$ ‰
E	leaf transpiration rate	mmol m^{-2} s^{-1}
g^t_{ac}	total conductance to CO_2 diffusion including boundary layer and stomatal conductance	mol m^{-2} s^{-1}
C_{ls}	leaf surface CO_2 mole fraction	µmol mol^{-1}
b_3	fractionation by Rubisco (Farquhar, 1983)	30 ‰
b_4	combined fractionation of the conversion of CO_2 to HCO_3^- and PEP carboxylation (Farquhar, 1983)	-5.74 ‰
e	fractionation factor associated with respiration (Pengelly et al., 2010)	$e = \delta^{13}\text{C in the CO}_2\text{cyllinder} - \delta^{13}\text{C in growth environment}$
g_m	mesophyll conductance to CO_2 (Ubierna et al., 2016)	g_m = 1.4 mol m^{-2} s^{-1} at 28 °C
Δ	Photosynthetic discrimination against ^{13}C	‰
O_m	O_2 mole fraction in the mesophyll cells	210000 µmol mol^{-1}
O_s	O_2 mole fraction in the bundle-sheath cells	$O_s = \frac{\alpha\cdot A}{0.047\cdot g_{bs}} + O_m$ µmol mol^{-1}
C_a	CO_2 mole fraction in the ambient air	Measured
C_s	CO_2 mole fraction in the bundle-sheath cells	$C_s = \frac{(\Gamma^*\cdot O_s)\cdot\left(\frac{7}{3}(A+R_d)+\frac{(1-x)\cdot J_t}{3}\right)}{\frac{(1-x)\cdot J_t}{3}-(A+R_d)}$ in µmol mol^{-1}
C_i	CO_2 mole fraction inside the leaf	µmol mol^{-1}
C_m	CO_2 mole fraction in the mesophyll cytosol at the sites of CA	$C_m = C_i - \frac{A}{g_m}$ in µmol mol^{-1}
R_d	non-photorespiratory CO_2 released in the dark (= measured rates of dark respiration)	µmol $m^{-2}s^{-1}$
R_m	Mesophyll mitochondrial respiration rate	$R_m = 0.5R_d$ in µmol $m^{-2}s^{-1}$
V_c	Rubisco carboxylation rate (von Caemmerer, 2000)	$V_c = \frac{(1-x)\cdot J_t}{3\cdot\left(1+\frac{7\cdot\Gamma^*\cdot O_s}{3\cdot C_s}\right)}$ in µmol $m^{-2}s^{-1}$
V_o	Rubisco oxygenation rate (von Caemmerer, 2000)	$V_o = \frac{V_c - A - R_d}{0.5}$ µmol $m^{-2}s^{-1}$
V_P	PEP carboxylation rate (von Caemmerer, 2000)	$V_p = \frac{x\cdot J_t}{2}$ µmol $m^{-2}s^{-1}$
α	fraction of PSII activity in the bundle-sheath cells (Sharwood et al., 2016)	0 for NADP-ME and 0.2 for NAD-ME and PEP-CK species
γ^*	half of the reciprocal of Rubisco specificity (Sharwood et al., 2016)	0.000255, 0.00023 and 0.000233 for NADP-ME, NAD-ME and PEP-CK species, respectively
Γ^* (µbar)	compensation point in the absence of mitochondrial respiration	$\Gamma^* = O_s \cdot \gamma^*$
