# Supplementary material for: Shade compromises the photosynthetic efficiency of NADP-ME less than that of PEP-CK and NAD-ME C4 grasses
Source: J Exp Bot. 2018 Apr 5;69(12):3053–68. doi: 10.1093/jxb/ery129 (PMC5972597; doi:10.1093/jxb/ery129)
Supplement: Supplementary Figures S1-S4 [file ery129_suppl_supplementary_figures_s1-s4.pdf]

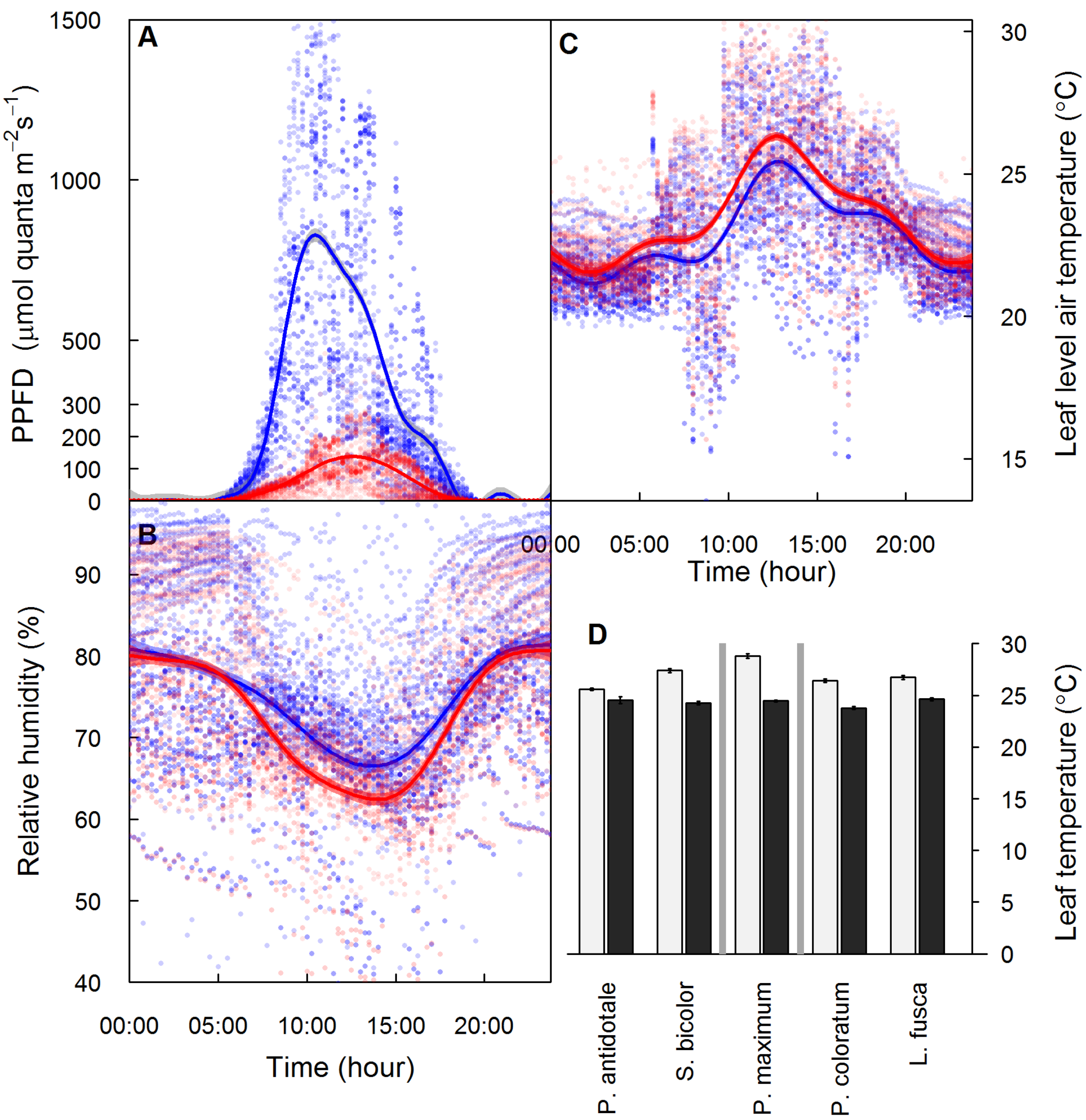

Fig. S1. Glasshouse growth conditions.
Glasshouse conditions during the experimental growth period; photosynthetic photon flux density, (A) PPFD, (B) relative humidity, and (C) and leaf-level air temperature. The solid lines represent the growth averages of data points. Average conditions for each treatment is also shown. (D) Instantaneous leaf temperature taken with an infrared camera (AGRI-THERM II™) of some grasses are shown during a typical sunny day for control (white columns) and shade (black columns) plants.

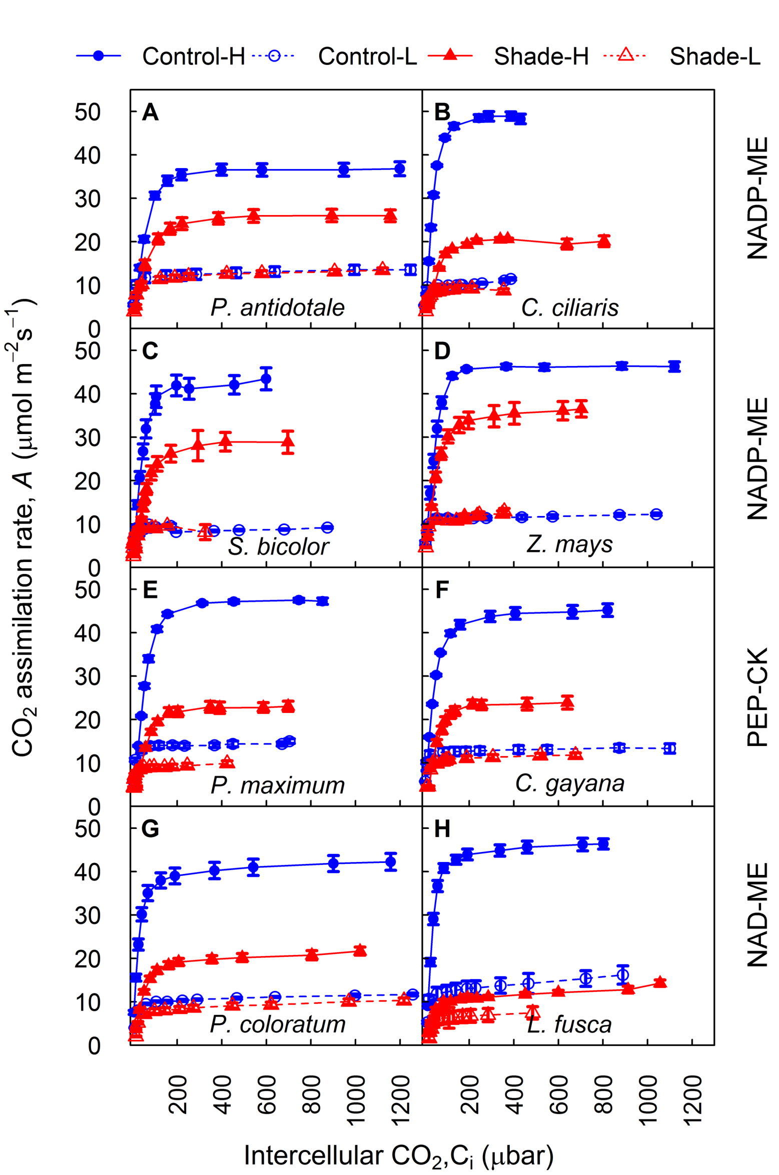

Fig. S2. Photosynthetic CO_2 response curves (*A*-C_i) of C_4 grasses.
Responses of CO_2 assimilation rate to increasing intercellular [CO_2], C_i were measured at low light, 250 μmol quanta m^{-2} s^{-1} (dashed lines and open symbols) and high light, 2000 μmol quanta m^{-2} s^{-1} (straight lines and closed symbols) in (A-D) C_4-NADP-ME, (E-F) C_4-PEP-CK and (G-H) C_4-NAD-ME grasses grown in control (full sunlight; blue and circles) or shade (16% of natural sunlight; red and triangles) environments. Values are means of 3-4 replicates ± SE.

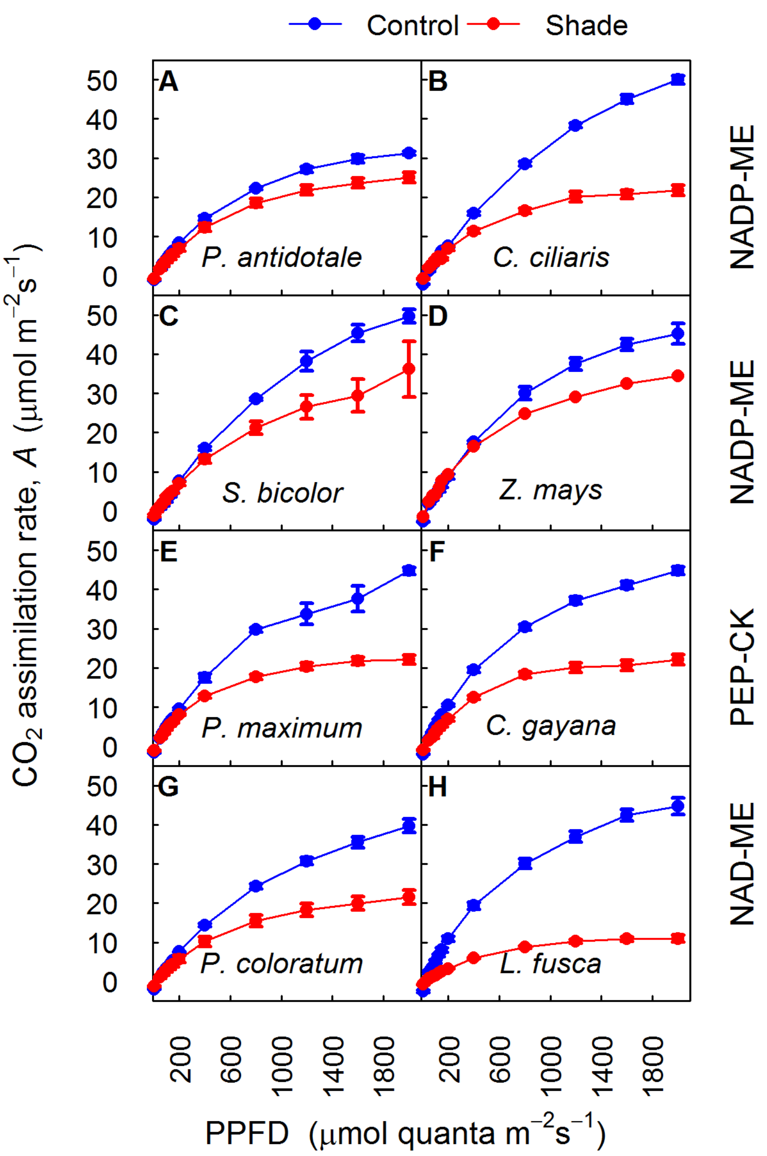

Fig. S3. Photosynthetic light response curves for C_4 grasses.
Responses of CO_2 assimilation rate (*A*) to increasing light (PPFD) were measured at saturating CO_2 (C_a = 650 µL L^{-1}) in (A-D) C_4-NADP-ME, (E-F) C_4-PEP-CK and (G-H) C_4-NAD-ME grasses grown in control (full sunlight; blue) or shade (16% of natural sunlight; red) environments. Values are means of 3-4 replicates ± SE

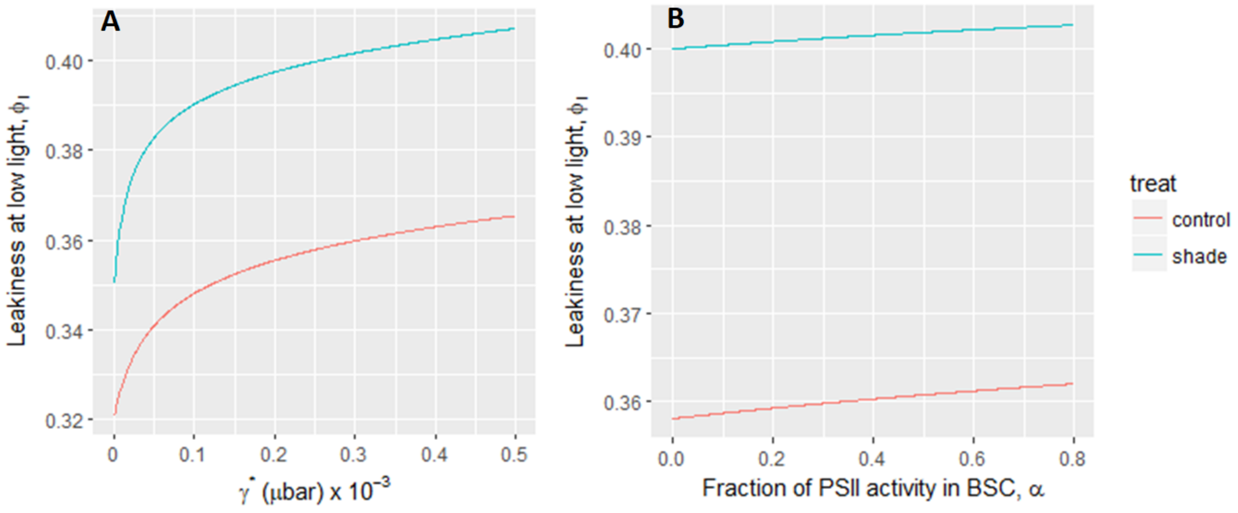

Fig. S4. Sensitivity of leakiness at low light to the (A) half of Rubisco specificity, γ* and (B) fraction of PSII in BSC, α, in *Z. mays* at low light (250 µmol quanta $m^{-2}s^{-2}$). The modelled line is solution of equation 9 for the range of γ* and α values shown on x-axis.
