# Supplementary material for: Shade compromises the photosynthetic efficiency of NADP-ME less than that of PEP-CK and NAD-ME C4 grasses
Source: J Exp Bot. 2018 Apr 5;69(12):3053–68. doi: 10.1093/jxb/ery129 (PMC5972597; doi:10.1093/jxb/ery129)
Supplement: Appendix S1 [file ery129_suppl_appendix_s1.pdf]

Appendix

Leakiness calculations at low light

The light limited C_4 photosynthesis model (von Caemmerer, 2000) was deployed to derive electron transport flux (J_t) as a function of photosynthetic rates (A) as described by Ubierna et al. (2013) as bellow,

$$J_\mathrm{t} = \frac{-II + \sqrt{II^2 - 4 \cdot III \cdot I}}{2 \cdot III} \qquad \text{(S1)}$$

where

$$I = \left(1 + \frac{R_\mathrm{d}}{A}\right) \cdot \left(R_\mathrm{m} - g_\mathrm{bs} \cdot c_\mathrm{m} \cdot \frac{7 \cdot g_\mathrm{bs} \cdot \Gamma^* \cdot O_\mathrm{m}}{3}\right) + (R_\mathrm{d} + A) \cdot \left(1 + \frac{7 \cdot \alpha \cdot \Gamma^*}{3 \cdot 0.047}\right) \qquad \text{(S2)}$$

$$II = \frac{1-x}{3} \cdot \left[\frac{g_\mathrm{bs}}{A} \cdot \left(c_\mathrm{m} - \frac{R_\mathrm{m}}{g_\mathrm{bs}} - \Gamma^* \cdot O_\mathrm{m}\right) - 1 - \frac{\alpha \cdot \Gamma^*}{0.047}\right] - \frac{x}{2} \cdot \left(1 + \frac{R_\mathrm{d}}{A}\right) \qquad \text{(S3)}$$

$$III = \frac{x - x^2}{6 \cdot A} \qquad \text{(S4)}$$

Variable definitions and units are presented in Table 1. We used measured values for α and Γ^* for biochemical subtypes of C_4 photosynthesis (Table 2, (Sharwood et al., 2016).

We calculate C_s (CO_2 mole fraction in the bundle-sheath, V_p (PEP carboxylation rate), V_c (Rubisco carboxylation rate) and V_o (Rubisco oxygenation rate) at low light using C_4 model (von Caemmerer, 2000)

$$C_\mathrm{s} = \frac{(\gamma^* \cdot O_\mathrm{s}) \cdot \left(\frac{7}{3} \cdot (A + R_\mathrm{d}) + \frac{(1-x) \cdot J_\mathrm{t}}{3}\right)}{\frac{(1-x) \cdot J_\mathrm{t}}{3} - (A + R_\mathrm{d})} \quad \text{(S5)}$$

$$V_\mathrm{p} = \frac{x \cdot J_\mathrm{t}}{2} \quad \text{(S6)}$$

$$V_\mathrm{c} = \frac{(1-x) \cdot J_\mathrm{t}}{3 \cdot \left(1 + \frac{7 \cdot \gamma^* \cdot O_\mathrm{s}}{3 \cdot C_\mathrm{s}}\right)} \quad \text{(S7)}$$

$$V_\mathrm{o} = \frac{V_c - A - R_\mathrm{d}}{0.5} \quad \text{(S8)}$$

The term $\overline{b_4}$ and $\overline{b_3}$ in Eqn 9 are defined as,

$$\overline{b_3} = b_3 - \frac{eR_\mathrm{d}}{V_\mathrm{c}} - \frac{0.5 \cdot f \cdot V_\mathrm{o}}{V_\mathrm{c}} \quad \text{(S9)}$$

$$\overline{b_4} = b_4 \left(1 - \frac{V_\mathrm{p}}{V_\mathrm{h}}\right) + (e_\mathrm{s} + h) \cdot \frac{V_\mathrm{p}}{V_\mathrm{h}} - \frac{eR_\mathrm{m}}{V_\mathrm{p}} \quad \text{(S10)}$$

and we assumed that CA is saturating and V_p/V_h is approx. 0, hence:

$$\overline{b_4} = b_4 - \frac{eR_\mathrm{m}}{V_\mathrm{p}} \quad \text{(S11)}$$

Note that equations 7 and 8 are rearranged forms of equations S9 and S10 from (von Caemmerer et al., 2014).
